# From scavenger to victim: mechanism and treatment of microglial ferroptosis in white matter injury disease

**DOI:** 10.3389/fimmu.2026.1859981

**Published:** 2026-09-30

**Authors:** Ruoyu Wang, Qiong Yang, Zheng Cao, Jianjie Zhou, Bingxin Tian, Xin Wang, Xiaohong Zhang, Qingwei Xiang, Gang Chen

**Affiliations:** 1 School of Basic Medical Sciences, Hubei University of Chinese Medicine, Wuhan, Hubei, China; 2 The First Clinical Medical School, Hubei University of Chinese Medicine, Wuhan, Hubei, China; 3 Department of Geriatrics, Hubei Provincial Hospital of Traditional Chinese Medicine, Wuhan, Hubei, China; 4 Hubei Shizhen Laboratory Department of Geriatrics & Department of Orthopedic Surgery, Wuhan, Hubei, China; 5 Hubei Provincial Hospital of Traditional Chinese Medicine (Affiliated Hospital of Hubei University of Chinese Medicine), Wuhan, Hubei, China; 6 The First People’s Hospital of Jingzhou (Jingzhou First People’s Hospital), Jingzhou, Hubei, China; 7 Xiangyang Hospital of Integrated Traditional Chinese and Western Medicine, Xiangyang, Hubei, China; 8 Hubei Provincial Academy of Traditional Chinese Medicine, Wuhan, Hubei, China; 9 Hubei Shizhen Laboratory, Wuhan, Hubei, China

**Keywords:** Alzheimer’s disease, ferroptosis, metabolic reprogramming, microglia, multiple sclerosis, phagocytic overload, vascular dementia, white matter injury

## Abstract

Failure of myelin repair is a shared pathological feature of white matter injury (WMI)-associated diseases, including Alzheimer’s disease (AD), vascular dementia (VaD), and multiple sclerosis (MS), but the underlying mechanisms remain incompletely understood. Microglia, the resident innate immune cells of the central nervous system (CNS), contribute to white matter homeostasis by clearing myelin debris and supporting remyelination. Within the iron- and lipid-rich white matter microenvironment, sustained uptake of myelin and oligodendroglial debris may impose phagocytic and metabolic stress on microglia, thereby increasing their susceptibility to ferroptosis, an iron-dependent form of regulated cell death driven by membrane phospholipid peroxidation. This susceptibility is regulated by interconnected mechanisms involving ferritinophagy-associated iron dysregulation, ACSL4/NOX2/15-LOX-related lipid peroxidation, and impaired endogenous ferroptosis-defense systems. Microglial responses are also heterogeneous: disease-associated, demyelination-associated, white matter-associated, lipid-droplet-accumulating, and degenerative microglial programs represent partially overlapping and context-dependent states rather than a proven linear trajectory. In susceptible microglial subsets, ferroptosis or ferroptosis-associated dysfunction may impair debris clearance and trophic support while promoting oxidized lipid accumulation and inflammatory signaling, thereby limiting remyelination. This review summarizes current evidence on the molecular mechanisms, multicellular interactions, microglial states, disease contexts, and therapeutic strategies associated with microglial ferroptosis in WMI. We further propose a functional therapeutic-window framework based on phagocytic competence, iron–lipid handling capacity, antioxidant reserve, and evidence of ferroptotic injury, and discuss major challenges in clinical translation. Overall, current evidence supports microglial ferroptosis as a context-dependent contributor to myelin repair failure rather than a universal determinant.

## 1 Introduction

White matter injury (WMI) is a shared pathological hallmark of Alzheimer’s disease (AD), vascular dementia (VaD), and multiple sclerosis (MS). Together, these disorders impose an enormous global health burden. AD is the leading cause of dementia worldwide. Its prevalence is projected to reach 153 million by 2050 (1). VaD, the second most common dementia subtype, is estimated to affect about 20.6 million individuals globally by the same year (2). MS currently affects about 1.89 million people worldwide, with roughly 62,000 new cases diagnosed annually (3). MS is also one of the principal causes of neurological disability among young and middle-aged adults (4). Current immunomodulatory and neuroprotective therapies can partially alleviate clinical symptoms. However, they do not halt WMI progression, and effective remyelination remains a major unmet clinical need.

Microglia are the resident innate immune cells of the central nervous system (CNS) (5). They are important regulators of white matter homeostasis (6). Under normal conditions, microglia facilitate remyelination (7). They clear inhibitory debris through phagocytosis (8). They also secrete neurotrophic factors that support the recruitment and differentiation of oligodendrocyte precursor cells (OPCs) (9). However, during sustained demyelination, this protective machinery becomes vulnerable to a pathological “metabolic trap.” Chronic demyelination may drive microglia toward a state of phagocytic overload (10). When intracellular processing capacity becomes insufficient, poorly processed lipids and redox-active iron may accumulate, thereby promoting lipid peroxidation and disrupting redox homeostasis (11–13).

Under these conditions, ferroptosis—an iron-dependent form of regulated cell death driven by membrane phospholipid peroxidation—may contribute to microglial dysfunction (14, 15). The development of ferroptosis-associated stress may represent a shift in microglial function from a neuroprotective scavenging state toward a dysfunctional state. This dysfunction may impair myelin debris clearance, increase the release of lipid peroxidation products and iron species, and contribute to the formation of a neurotoxic microenvironment (16). Through ferroptosis-associated inflammatory signaling, microglia under ferroptosis-associated stress may further contribute to a positive feedback loop between ferroptosis-associated stress and neuroinflammation that can affect oligodendrocyte integrity and OPC differentiation (17). Thus, microglial ferroptosis may represent an important contributor to remyelination failure under selected pathological conditions (18).

The susceptibility of white matter microglia to ferroptosis is likely shaped by the interaction among autophagy–lysosomal dysfunction, iron dyshomeostasis, lipid peroxidation, and impaired endogenous anti-ferroptotic defenses. These processes are closely coupled with microglial metabolic reprogramming but should not be interpreted as a fixed or universal sequence. Given the role of microglia in myelin surveillance, debris clearance, and metabolic regulation, targeting microglial ferroptosis and preserving adaptive microglial functions may offer therapeutic potential in WMI-associated diseases. This review proposes a conceptual framework in which microglial ferroptosis is considered a context-dependent immunometabolic mechanism in WMI. We summarize current evidence regarding the molecular regulatory networks governing susceptibility to ferroptosis and their dynamics across AD, VaD, and MS. This framework offers a common pathological underpinning of WMI-associated diseases and provides a conceptual basis for developing clinical strategies to mitigate WMI and promote neural repair.

## 2 Multicellular and neurovascular regulation of white matter homeostasis and ferroptosis susceptibility

### 2.1 White matter structural and metabolic characteristics

Iron distribution within the brain exhibits marked regional heterogeneity (19). *In vivo* magnetic resonance imaging (MRI) studies have demonstrated that, although overall iron concentrations in white matter are lower than those in deep gray matter in the healthy adult brain, substantial iron accumulation persists in specific white matter tracts, including the corpus callosum and internal capsule (20).

This distribution pattern is closely associated with the unique cellular and structural characteristics of white matter (21). White matter is primarily composed of highly organized myelinated axons (22), structurally supported by lipid-rich myelin sheaths and the oligodendrocytes responsible for their formation (7). As the predominant iron-enriched cells in the CNS, oligodendrocytes maintain relatively high intracellular iron levels (23), thereby contributing to local iron enrichment in white matter.

Moreover, the biochemical composition of myelin further increases its susceptibility to oxidative damage. Myelin sheaths are enriched in lipids (24), particularly cholesterol and phospholipids, forming a dense lipid reservoir (25–28). Polyunsaturated fatty acids (PUFAs) within myelin-associated glycerophospholipids are especially prone to lipid peroxidation (29–31). In parallel, iron serves as a critical cofactor for enzymes involved in myelin biosynthesis (32). In white matter, iron is primarily associated with oligodendroglial metabolism, ferritin storage, cellular transport systems, and myelin maintenance rather than being freely stored within intact myelin structures (33).

Under pathological conditions, myelin and oligodendroglial injury disrupt white matter iron and lipid homeostasis, exposing microglia to sustained myelin-derived lipid substrates and altered iron availability. These combined metabolic stresses promote lipid peroxidation and increase microglial susceptibility to ferroptosis. Microglia may be particularly susceptible to ferroptosis-associated stress under specific pathological conditions (34). Acyl-CoA synthetase long-chain family member 4 (ACSL4) is a key determinant of this vulnerability, as it drives ferroptotic processes by regulating PUFA metabolism and promoting lipid peroxidation (35–37). Inhibition of ACSL4 expression has been shown to effectively attenuate HIV-1 Tat–induced ferroptosis and associated inflammatory responses in microglia (38). However, its role in certain ischemic models remains controversial (37). In addition, the identification of the novel susceptibility gene SEC24 homolog B, COPII coat complex component (SEC24B) provides a potential new target for modulating microglial ferroptosis (39).

### 2.2 Multicellular and neurovascular maintenance of white matter homeostasis

White matter homeostasis is maintained through coordinated interactions among vascular, glial, and neuronal compartments rather than through the function of any single cell population (7, 40–42). Brain endothelial cells and pericytes provide the vascular foundation of this network by preserving blood–brain barrier (BBB) integrity, regulating capillary stability, and controlling the delivery of oxygen and metabolic substrates. In addition to their vascular functions, endothelial-derived signals contribute to oligovascular coupling and regulate oligodendroglial responses; for example, endothelial caveolin-1 modulates vascular heat shock protein 90 alpha (HSP90α) signaling, endothelial–OPC communication, and oligodendrogenesis during chronic cerebral ischemia (40).

Astrocytes serve as a critical metabolic and regulatory interface between blood vessels and neural cells through their perivascular endfeet. They participate in neurovascular coupling, nutrient exchange, ion homeostasis, and redox regulation, thereby contributing to the maintenance of the extracellular environment required for white matter integrity (41). Astrocyte–oligodendrocyte interactions further support lipid metabolism and remyelination through coordinated regulation of cholesterol metabolism and antioxidant pathways (42).

Oligodendrocytes and axons maintain a reciprocal metabolic relationship that is essential for long-term white matter stability. Oligodendrocytes provide the metabolic support necessary for axonal maintenance, whereas neuronal activity influences oligodendroglial metabolic adaptation and myelin remodeling (43).

Microglia represent an additional regulatory component within this multicellular network by continuously surveying the white matter microenvironment and contributing to myelin maintenance. Beyond immune surveillance, microglia regulate myelin growth, turnover, and oligodendroglial lipid metabolism; selective microglial depletion disrupts adult myelin maintenance and promotes white matter degeneration (7).

Collectively, vascular regulation, astrocyte-mediated metabolic support, oligodendrocyte–axon coupling, and microglial surveillance form an interconnected cellular network that preserves white-matter structure, metabolic homeostasis, and functional integrity.

### 2.3 Convergent disruption by demyelination and neurovascular dysfunction

#### 2.3.1 Demyelination and neurovascular dysfunction as interacting pathological inputs

Demyelination and neurovascular dysfunction represent two interacting pathological processes that converge to disrupt white matter homeostasis. Demyelination involves not only the loss of myelin structures but also oligodendrocyte dysfunction, axonal metabolic stress, and the accumulation of lipid-rich myelin debris (43–46). Impaired myelin turnover increases the demand for efficient phagocytic clearance, whereas disruption of microglia–oligodendrocyte communication may compromise the resolution of myelin damage and subsequent repair processes (47–49).

Neurovascular dysfunction contributes to white matter injury through impaired perfusion, endothelial dysfunction, and blood–brain barrier disruption. Chronic cerebral hypoperfusion induces vascular abnormalities, oxidative stress, and glial activation before the development of overt white matter degeneration, suggesting that vascular insufficiency may serve as an early driver of metabolic stress within the white matter microenvironment (50, 51). Furthermore, endothelial caveolin-1 deficiency disrupts endothelial–OPC communication and impairs oligodendrogenesis following ischemic injury, highlighting the importance of vascular-derived signals in maintaining oligodendroglial repair capacity (40).

These pathological processes are not independent but may amplify each other through reciprocal interactions between vascular, glial, and neuronal compartments. Reduced vascular support increases oligodendrocyte vulnerability and limits regenerative capacity, whereas persistent myelin degeneration may further disrupt vascular–glial communication and exacerbate tissue stress (40, 51, 52). Thus, demyelination and neurovascular dysfunction converge to reshape the white matter microenvironment, creating conditions that favor metabolic dysregulation, oxidative stress, and impaired tissue repair.

#### 2.3.2 Remodeling of the iron–lipid microenvironment

Demyelination and vascular dysfunction reshape the white matter microenvironment by disrupting lipid metabolism and iron homeostasis (13, 18). Because myelin is highly enriched in cholesterol, phospholipids, and polyunsaturated fatty acids, myelin degeneration results in the release of abundant membrane-derived lipids, including oxidized lipid species generated under inflammatory and oxidative conditions (46, 53).

Iron homeostasis is similarly remodeled after white matter injury. Demyelination-associated iron is unlikely to be released predominantly as freely diffusible Fe^3+^ or Fe^2+^. Instead, it is carried mainly within ferritin, iron-containing proteins, and oligodendroglial cellular or organellar remnants admixed with fragmented myelin (13, 54, 55). Human aging white matter lesions contain myelin-laden, ferritin light chain (FTL)-enriched microglia, supporting the concept that phagocytes acquire ferritin-associated iron together with degenerating myelin and oligodendroglial debris (13). Following phagocytic uptake, lysosomal degradation can liberate protein-bound iron, which may be resequestered in ferritin, exported, or incorporated into lysosomal and cytosolic labile Fe^2+^ pools; increases reactive oxygen species (ROS) generation and membrane phospholipid peroxidation (56, 57).

In contrast, neurovascular dysfunction primarily affects the regulation and distribution of circulating transferrin-bound Fe^3+^ rather than generating an independent iron source. Under physiological conditions, brain endothelial cells regulate transvascular iron transport and contribute to iron delivery within the CNS. However, hypoperfusion and increased uptake of myelin-derived debris may disturb endothelial iron handling, promote endothelial ferritin and labile iron accumulation, impair transferrin transport, and reduce the iron availability required for oligodendroglial maintenance and remyelination (58, 59). When BBB disruption is accompanied by microhemorrhage or substantial blood extravasation, hemoglobin- and heme-bound iron provide additional sources of iron, and subsequent heme degradation may release redox-active Fe^2+^.

The coexistence of excessive lipid substrates and disrupted iron regulation creates a microenvironment that favors iron-dependent lipid peroxidation. Experimental demyelination is associated with increased iron deposition, enhanced lipid peroxidation, and reduced antioxidant capacity, suggesting that susceptibility to ferroptosis arises from the coordinated disruption of iron–lipid homeostasis rather than from a single molecular abnormality (13, 18, 60). Within this metabolically stressed environment, microglia become critical responders because they are responsible for processing iron- and lipid-rich myelin debris. However, persistent metabolic burden may eventually impair microglial lipid handling, iron regulation, and protective functions, thereby contributing to white matter injury progression (18, 46, 53).

#### 2.3.3 Microglial integration of phagocytic and metabolic stress

Microglia serve as a critical link between extracellular white matter injury and intracellular metabolic adaptation through the phagocytosis of myelin and oligodendroglial debris (61, 62). Following phagocytic uptake, myelin-derived cholesterol, phospholipids, sphingolipids, proteins, and iron-containing cellular components are transported to phagolysosomal compartments for processing, after which their constituents may undergo recycling, storage, metabolic utilization, or export (62, 63).

Under adaptive conditions, enhanced lysosomal processing, peroxisomal lipid catabolism, cholesterol efflux, ferritin-mediated iron sequestration, and antioxidant responses enable microglia to efficiently remove inhibitory debris while limiting lipotoxicity and oxidative damage (46, 53, 62, 63). Supporting this concept, microglial peroxisomal biogenesis factor 5 (PEX5) deficiency impairs lipophagy and lysosomal integrity, promotes lipid droplet accumulation, and disrupts debris clearance and remyelination (53). These findings highlight the importance of intracellular lipid processing pathways in maintaining the beneficial functions of microglia during myelin remodeling.

However, this adaptive response may become maladaptive when persistent substrate influx exceeds intracellular processing capacity (61, 62). Impairment of lysosomal degradation, fatty-acid utilization, cholesterol export, iron sequestration, or iron efflux may promote the accumulation of incompletely processed lipids and redox-active iron. Nuclear factor erythroid 2-related factor 2 (Nrf2)-dependent metabolic reprogramming facilitates lipid and iron handling and supports remyelination, whereas insufficient Nrf2 activity is associated with iron retention, increased phospholipid peroxidation, and enhanced susceptibility to ferroptosis (18). Lipid droplets should therefore be interpreted within their biological context: transient lipid droplet formation may serve as an adaptive mechanism to sequester excess fatty acids, whereas persistent accumulation accompanied by impaired turnover and oxidative stress may reflect declining metabolic resilience (63, 64).

Thus, myelin phagocytosis itself is not intrinsically ferroptotic. Rather, susceptibility to ferroptosis is likely to emerge when sustained debris uptake converges with lysosomal dysfunction, impaired lipid disposal, expansion of labile iron pools, and insufficient antioxidant defenses (18, 46, 53, 61–63). Recent experimental evidence provides more direct support for this model. Myelin-loaded primary microglia exhibit lipid and Fe^2+^ accumulation, mitochondrial oxidative stress, and membrane lipid peroxidation, with cytotoxicity preferentially rescued by ferrostatin-1 and deferoxamine. Mechanistically, ERK5 promotes ferroptosis through NFATc4-dependent induction of the lipid scramblase CLPTM1L, whereas ERK5 inhibition reduces iron and lipid oxidative stress. Consistently, pharmacological or microglia-specific genetic inhibition of ERK5 attenuates white matter injury and improves cognitive function *in vivo* (65). Human white matter lesions containing myelin-laden microglia with iron–lipid imbalance, mitochondrial abnormalities, and DNA damage provide evidence supporting the pathological relevance of this metabolic failure. These findings strengthen the concept that persistent myelin processing can become a source of iron–lipid metabolic stress when intracellular adaptive capacity is exceeded, thereby linking phagocytic overload to microglial ferroptosis. However, these findings should be interpreted as indicators of ferroptosis-associated cellular stress or increased susceptibility rather than as independent proof of ferroptotic cell death (**Figure 1**).

**Figure 1:**
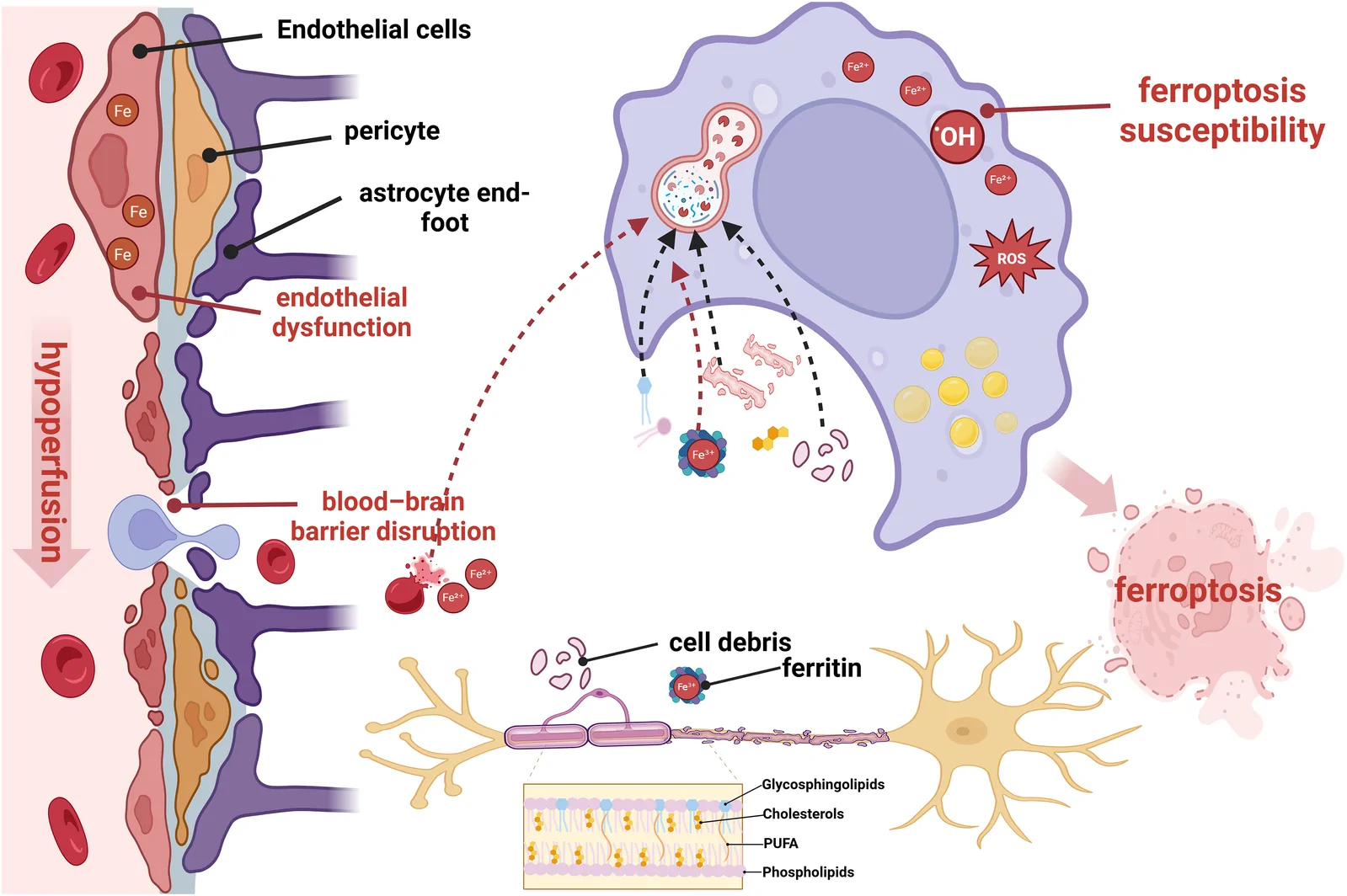
Convergent effects of demyelination and neurovascular dysfunction on the white matter iron–lipid microenvironment and microglial metabolic stress. Demyelination promotes the accumulation of lipid-rich myelin debris, oligodendrocyte dysfunction, and axonal metabolic stress, whereas neurovascular dysfunction impairs cerebral perfusion, endothelial signaling, blood–brain barrier integrity, and iron transport. Together, these processes disrupt iron and lipid homeostasis within white matter and increase the phagocytic and metabolic burden on microglia. Under adaptive conditions, lysosomal processing, lipid catabolism, cholesterol efflux, ferritin-mediated iron sequestration, iron export, and antioxidant responses support debris clearance and remyelination. When persistent substrate influx exceeds intracellular processing capacity, defective lipid disposal, iron retention, expansion of labile Fe^2+^ pools, and oxidative stress promote microglial metabolic decompensation and increase susceptibility to ferroptosis. Myelin phagocytosis itself is not intrinsically ferroptotic, and iron–lipid accumulation, oxidative stress, or other stress-associated alterations in human white matter lesions should be interpreted as indicators of increased susceptibility to ferroptosis rather than independent evidence of ferroptotic cell death. BBB, blood–brain barrier; Fe^2+^, ferrous iron. Created with BioRender.com.

### 2.4 Regional context: white matter versus gray matter microglial responses

White matter microglia are specialized by their continuous interaction with myelin-rich structures. Aging and demyelination induce microglial states enriched in phagocytosis, lysosomal processing, and lipid metabolism programs (66). Human white matter lesions contain microglia with iron accumulation, lipid droplets, and ferroptosis-associated alterations, suggesting that sustained processing of lipid- and iron-rich debris contributes to susceptibility to ferroptosis (13).

In gray matter, microglial metabolic stress is more closely associated with neuronal dysfunction and proteinopathy-related signals. Tauopathy and Alzheimer’s disease models demonstrate that neuronal lipid dysregulation and APOE4-associated pathways promote microglial lipid droplet accumulation and inflammatory activation (64, 67). These processes may increase oxidative stress, although lipid droplet accumulation itself does not establish ferroptosis.

White matter and gray matter microglia share common ferroptotic mechanisms involving iron accumulation, phospholipid peroxidation, and impaired antioxidant defense. However, white matter microglia are primarily challenged by myelin-derived lipid and iron burdens, whereas gray matter microglia are exposed to neuron-derived lipid stress and proteinopathy-associated metabolic dysfunction. These differences reflect regional adaptations rather than distinct ferroptosis pathways.

### 2.5 Microglia as an integrative node within the multicellular white matter network

Ferroptotic susceptibility in white matter involves multiple cell populations. Oligodendrocytes are vulnerable because iron-dependent metabolism and lipid synthesis are essential for myelin maintenance (44, 60). OPCs undergo ferroptotic injury after heme exposure, contributing to impaired remyelination after hemorrhagic injury. Astrocytes regulate antioxidant capacity and lipid metabolism through glutathione and Nrf2 pathways (42). Endothelial dysfunction alters vascular support and barrier properties, while axons remain dependent on oligodendroglial metabolic coupling (40, 43, 68).

Microglia are emphasized not because they are the only ferroptosis-sensitive cells, but because they serve as an integrative interface connecting signals from oligodendrocytes, axons, astrocytes, and the neurovascular unit. Through immune surveillance, debris clearance, and regulation of iron, lipid, and inflammatory pathways, microglia integrate diverse environmental cues within white matter (7, 62). Single-cell studies show that white matter injury induces microglial programs associated with phagocytosis, lysosomal activity, and lipid metabolism, highlighting their role in tissue adaptation (66). Microglia may therefore function as an important regulatory node that influences whether multicellular white matter stress is resolved through repair or progresses toward persistent tissue injury. This context-dependent role provides the basis for examining the microglial states that emerge under WMI-associated stress.

## 3 Triggering mechanisms: context-dependent microglial states and metabolic reprogramming

### 3.1 Principles for classifying microglial populations and states

Microglial nomenclature has become increasingly complex and currently encompasses several distinct dimensions, including anatomical localization, pathological context, transcriptional characteristics, metabolic phenotype, and cell-death status (69). White matter-resident and gray matter-resident microglia refer primarily to microglial populations defined according to their regional localization within the central nervous system. In contrast, disease-associated microglia (DAM) (70) and demyelination-associated microglia (DMAM) (71, 72) describe context-dependent activation programs that arise predominantly in response to neurodegenerative and demyelinating pathological conditions, respectively. White matter-associated microglia (WAM) represent a transcriptionally and functionally distinct microglial state that is preferentially enriched in aging or degenerating white matter (66). Therefore, WAM should not be regarded as equivalent to all microglia residing in white matter regions. Lipid-droplet-accumulating microglia (LDAM) are characterized by excessive intracellular lipid accumulation and impaired lipid metabolism, whereas degenerative microglia exhibit a more severe stress-associated phenotype involving iron–lipid dyshomeostasis, mitochondrial abnormalities, and DNA damage (64). Ferroptotic microglia, by comparison, refer to microglial cells undergoing iron-dependent cell death driven by membrane phospholipid peroxidation rather than to a stable transcriptional subtype (13). It should be noted that these categories describe different, although partially overlapping, aspects of microglial identity and functional status. Thus, they should not be organized into a fixed hierarchical framework or interpreted as successive stages of a universal microglial lineage.

### 3.2 DAM and DMAM: disease- and demyelination-associated programs

Disease-associated microglia (DAM) are primarily associated with neurodegenerative pathology (70, 73). Human single-nucleus studies have identified distinct amyloid-β- and tau-associated microglial populations, indicating that microglial responses are heterogeneous and dependent on the underlying pathological context (74). DAM commonly exhibit reduced expression of the homeostatic genes P2RY12, TMEM119, and CX3CR1, together with increased expression of APOE, LPL, CST7, and AXL. These molecular changes reflect enhanced phagocytosis, lysosomal activity, lipid handling, and inflammatory signaling (70, 73). TREM2 contributes to lipid sensing, plaque association, and microglial adaptation to neurodegeneration-associated substrates (75, 76). These responses may initially facilitate the clearance of pathological substrates. However, persistent stimulation or ineffective lipid processing may promote sustained microglial activation and chronic inflammation (62, 73).

Demyelination-associated microglia (DMAM), in contrast, arise during active demyelination, when microglia are required to clear myelin debris and support remyelination (53, 77, 78). In cuprizone models, DMAM show increased expression of Ms4a family members, Apoe, Gpnmb, Abca1, and other genes involved in lipid processing and lysosomal function. Microglia-specific PEX5 deletion increases lipid droplet accumulation, impairs lipophagy and lysosomal integrity, and consequently disrupts myelin debris clearance and remyelination. Thus, although DAM and DMAM share phagocytic, lysosomal, and lipid-metabolic modules, they arise in response to distinct pathological pressures. They should therefore not be regarded as identical populations or interpreted as sequential states within a universal microglial trajectory.

### 3.3 WAM: a white-matter-enriched state

Single-cell transcriptomic profiling has identified white matter-associated microglia (WAM) as an age-dependent microglial state that is preferentially enriched in white matter regions (66). WAM share certain molecular and functional features with DAM, including the activation of phagocytic and lipid metabolic programs; however, they can be distinguished by their regional enrichment, close association with degenerating myelin, and adaptation to the unique microenvironment of aging white matter (66). In aged white matter, WAM accumulate around damaged myelin structures and are considered to participate in myelin debris clearance and tissue maintenance (61, 66). The generation and functional adaptation of WAM are dependent on TREM2 signaling, highlighting the importance of lipid sensing and microglial metabolic regulation in age-associated white matter remodeling (62, 66).

However, it should be noted that WAM do not encompass all white matter-resident microglia and should not be considered established downstream derivatives of DAM or DMAM (66). The molecular similarities between these states are more likely to reflect the convergent activation of shared functional programs, including lipid sensing, phagocytosis, and lysosomal processing, in response to distinct pathological substrates and microenvironmental cues. Current evidence supports an initially adaptive role of WAM in maintaining tissue homeostasis and facilitating debris clearance. However, whether persistent metabolic stress drives WAM-like cells toward metabolic decompensation or functional exhaustion remains to be determined (61–63).

### 3.4 LDAM: lipid storage and metabolic dysfunction

Lipid-droplet-accumulating microglia (LDAM) are characterized by increased lipid droplet accumulation, impaired lipid handling, oxidative stress, inflammatory activation, and reduced homeostatic functions (64). Recent studies in human Alzheimer’s disease (AD) have identified plaque-associated lipid-laden microglia and an APOE4/4-associated ACSL1-high microglial state, which is linked to enhanced triglyceride synthesis, lipid droplet formation, and potentially neurotoxic responses.

However, lipid droplet formation itself is not necessarily indicative of a pathological state (63). Under certain conditions, lipid droplets may represent an adaptive metabolic response by sequestering excess fatty acids and reducing their incorporation into highly oxidizable membrane phospholipids (63). Therefore, the biological consequences of lipid droplet accumulation are likely determined by multiple factors, including the duration of accumulation, lipid composition, turnover capacity, and the efficiency of lipid metabolic pathways such as lipophagy, fatty acid oxidation, and cholesterol efflux (62, 63). LDAM may acquire metabolic characteristics that increase susceptibility to ferroptosis under persistent lipid and oxidative stress. Nevertheless, lipid droplet accumulation alone does not demonstrate membrane phospholipid peroxidation or establish ferroptotic cell death.

### 3.5 Degenerative microglia in human white matter injury

Adeniyi et al. identified degenerative microglia in aging human white matter lesions associated with Alzheimer’s disease and vascular dementia (13). These microglial populations were enriched in white matter hyperintensities and exhibited features of cellular stress, including FTL accumulation, perilipin 2 (PLIN2)-positive lipid droplets, phosphorylated histone H2AX (γH2AX)-associated DNA damage, and translocase of the outer mitochondrial membrane 20 (TOM20)-related mitochondrial abnormalities. Their close association with myelin loss suggests the presence of profound iron–lipid dyshomeostasis and ferroptosis-associated cellular stress within vulnerable white matter microenvironments (13).

However, these molecular and pathological features should not be considered independent evidence of ferroptotic cell death (13, 39). In addition, post-mortem analyses provide limited information regarding the temporal relationship between ferroptosis-associated stress and degenerative microglial states. It remains unclear whether ferroptosis-associated stress represents an initiating event that contributes to microglial degeneration, a consequence of metabolic dysfunction, or a feature restricted to a specific vulnerable cellular subset. Therefore, degenerative microglia should be interpreted as a ferroptosis-associated pathological phenotype characterized by cellular stress and metabolic dysfunction rather than as an obligatory terminal derivative of LDAM (13, 39) (**Figure 2**).

**Figure 2:**
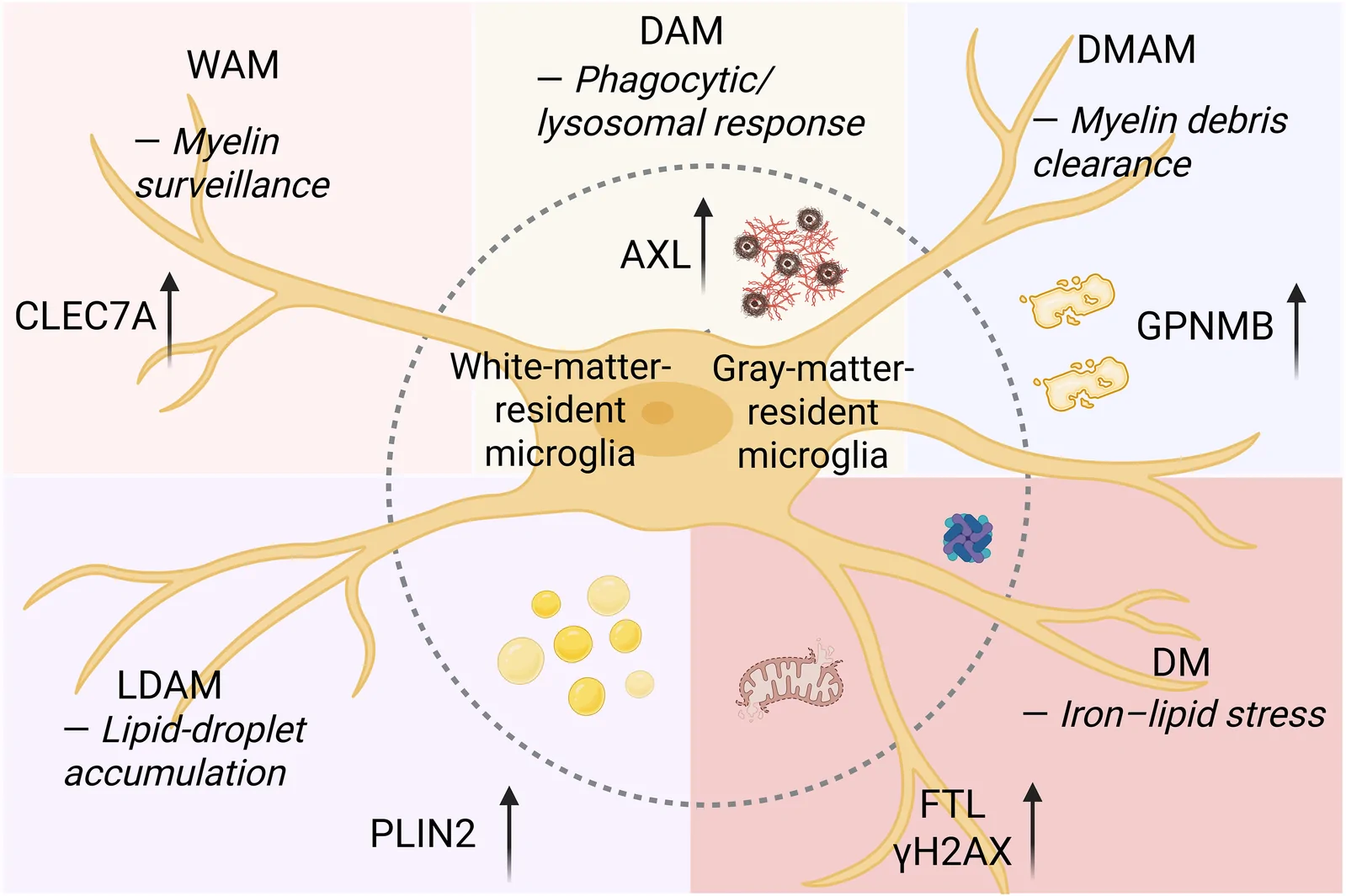
Context-dependent microglial states in white matter injury. Microglial heterogeneity in white matter injury reflects differences in anatomical localization, pathological context, transcriptional programs, and metabolic state. WAM, DAM, DMAM, LDAM, and degenerative microglia represent partially overlapping, context-dependent programs rather than fixed sequential states. Representative features shown include CLEC7A-associated myelin surveillance in WAM, AXL-associated phagocytic/lysosomal responses in DAM, GPNMB-associated myelin-debris processing in DMAM, PLIN2-associated lipid-droplet accumulation in LDAM, and FTL/γH2AX-associated iron–lipid stress and cellular damage in degenerative microglia. These markers are illustrative and are not exclusive identifiers of individual states. Current evidence does not support a universal hierarchical or linear transition among these microglial programs. DAM, disease-associated microglia; DMAM, demyelination-associated microglia; LDAM, lipid-droplet-accumulating microglia; WAM, white matter-associated microglia. Created with BioRender.com.

### 3.6 Relationship among microglial states and ferroptosis

DAM, DMAM, WAM, LDAM, and degenerative programs represent partially overlapping and context-dependent microglial states identified across different anatomical regions and pathological conditions (13, 53, 64, 67, 70, 79). Although these states exhibit distinct molecular signatures, shared expression of genes involved in phagocytosis, lysosomal activity, lipid metabolism, and stress responses likely reflects convergent functional adaptation to diverse pathological stimuli rather than direct lineage relationships.

The relationship between microglial states and ferroptosis is likely multifaceted. Several potential interactions may exist between microglial state variation and susceptibility to ferroptosis (13, 18, 39, 80). First, specific microglial states may increase susceptibility to ferroptosis by promoting iron retention, accumulation of oxidizable lipids, lysosomal dysfunction, or impaired antioxidant capacity (13, 18, 39, 64). Second, sublethal lipid peroxidation may function as an upstream stress signal that reshapes microglial transcriptional programs and functional states before the occurrence of overt cell death. Third, microglial state remodeling and ferroptosis may occur concurrently as parallel consequences of shared pathological stressors, including aging, hypoperfusion, amyloid-β accumulation, demyelination, and chronic inflammation. Fourth, ferroptotic cells may represent only a vulnerable subset within a transcriptionally defined microglial population, whereas other cells within the same state may remain adaptive, metabolically stressed, or functionally viable (13, 39, 64). Current evidence does not show which microglial state is most susceptible to ferroptosis. DAM-, DMAM-, and WAM-like microglia may remain adaptive when they can efficiently process phagocytosed material and maintain metabolic balance. In contrast, LDAM and degenerative microglia often show lipid accumulation, iron–lipid imbalance, oxidative stress, and mitochondrial dysfunction. These features may increase susceptibility to ferroptosis, but they do not by themselves prove ferroptotic cell death. TREM2/APOE signaling may also influence this vulnerability by regulating lipid sensing and metabolic adaptation. Overall, susceptibility to ferroptosis may depend more on phagocytic burden, iron and lipid handling, and antioxidant capacity than on microglial state alone.

Cell-specific studies further support a causal contribution of microglial ferroptosis in selected WMI contexts, including experimental evidence that modulation of Nrf2- and ERK5-related pathways can reduce ferroptotic injury and improve white matter outcomes (18, 80). These findings highlight the pathological relevance of microglial ferroptosis in white matter injury; however, they do not establish a fixed DAM–WAM–LDAM–degenerative sequence or demonstrate that specific transcriptional states inevitably progress toward ferroptotic death.

Therefore, iron accumulation, lipid droplet formation, oxidative stress, and ferroptosis-associated gene signatures should be interpreted as indicators of pathway activation or increased cellular susceptibility rather than direct evidence of ferroptotic cell death (13, 39, 80). These observations shift the focus from microglial state labels to the molecular determinants of ferroptotic susceptibility, which are discussed below (**Table 1**).

**Table 1:** Context-dependent microglial programs relevant to iron–lipid dysregulation and susceptibility to ferroptosis.

Microglial program/state	Typical context	Representative features	Functional interpretation	Relationship to ferroptosis	Refs.
DAM	Neurodegenerative proteinopathy, particularly AD	Reduced homeostatic markers; increased APOE, AXL, CST7, SPP1, and phagocytic/lysosomal programs; often TREM2 linked.	Context-dependent response to aggregated proteins and damaged tissue; may initially support clearance but become maladaptive with persistent stimulation.	May alter iron–lipid handling and antioxidant reserve, but DAM identity alone does not indicate ferroptotic death.	(62, 69, 72–75)
DMAM	Active demyelination and remyelination models	MS4A family, APOE, GPNMB, ABCA1; enhanced lysosomal and lipid-processing programs.	Supports myelin debris clearance and repair when processing capacity is preserved; PEX5 loss causes lipophagy failure and remyelination defects.	Persistent myelin-derived lipid and iron burden may increase ferroptotic susceptibility; direct execution evidence remains limited.	(53, 70, 71, 76, 77)
WAM	Aging or degenerating white matter	Regional enrichment in white matter; association with damaged myelin; TREM2-dependent phagocytic and lipid-metabolic programs.	Initially adaptive state involved in myelin surveillance and debris clearance.	Not a universal precursor of LDAM or degenerative microglia; progression toward ferroptotic injury is unproven.	(61–63, 65)
LDAM	Aging and neurodegenerative disease; APOE4-associated lipid stress	Lipid droplets, impaired lipid turnover, oxidative stress, inflammatory activation, reduced homeostatic function; ACSL1-high programs in selected contexts.	Lipid storage may be adaptive when turnover remains intact but maladaptive when lipophagy, fatty acid oxidation, or cholesterol efflux fail.	May create a ferroptosis-prone metabolic state; lipid droplets alone are not evidence of ferroptosis.	(62–64, 66)
Degenerative microglia	Human aging white matter lesions, AD/VaD-associated WMHs	FTL accumulation, PLIN2-positive lipid droplets, γH2AX-associated DNA damage, TOM20 abnormalities, and myelin loss.	Severe stress-associated and metabolically dysfunctional phenotype in vulnerable white matter.	Best described as ferroptosis-associated or ferroptosis-prone unless cell death, iron dependence, phospholipid peroxidation, and rescue are demonstrated.	(13, 39)

These programs are partially overlapping. AD, Alzheimer’s disease; DAM, disease-associated microglia; DMAM, demyelination-associated microglia; LDAM, lipid-droplet-accumulating microglia; VaD, vascular dementia; WAM, white matter-associated microglia; WMH, white matter hyperintensity.

## 4 Microglial susceptibility to ferroptosis: integrated regulatory networks

### 4.1 Ferritinophagy and iron metabolism imbalance

Cellular iron homeostasis is maintained through coordinated iron uptake, utilization, storage, and export (81, 82). Iron enters cells mainly as transferrin-bound iron (TBI) or non-transferrin-bound iron (NTBI) (83). TBI is internalized through transferrin receptor 1 (TfR1), reduced by six-transmembrane epithelial antigen of prostate 3 (STEAP3), and transported into the cytosol by divalent metal transporter 1 (DMT1), whereas NTBI contributes more prominently during iron overload (84–90). Cytosolic iron is then either used for heme, iron–sulfur cluster synthesis, and other iron-dependent processes or stored in ferritin to limit iron toxicity (91–99). Excess iron is exported by ferroportin, which is negatively regulated by hepcidin (100–103). The iron-responsive element/iron-regulatory protein (IRE/IRP) system further maintains iron balance by adjusting TfR1 and ferritin expression according to intracellular iron levels (104–106). When these regulatory mechanisms are overwhelmed, labile Fe^2+^ accumulates and promotes Fenton chemistry, lipid peroxidation, and ferroptosis (107–109).

Within the CNS, microglia exhibit a distinctive iron metabolism phenotype that differs substantially from that of neurons and other glial cells, forming an important cellular basis of WMI pathology (15, 18, 19, 110–113). With respect to iron uptake, pro-inflammatory stimuli — including lipopolysaccharide (LPS), amyloid-β (Aβ), and interferon-γ (IFN-γ) — upregulate the expression of TfR1, DMT1, and heme oxygenase-1 (HO-1), collectively promoting the preferential uptake of NTBI (114–116). Concomitantly, the phagocytosis of myelin debris further augments intracellular iron loading, resulting in a pronounced expansion of the labile iron pool. Iron efflux capacity in microglia is, by contrast, relatively constrained. Under inflammatory and aging conditions, hepcidin has been shown to induce the endocytic internalization and lysosomal degradation of the membrane iron exporter ferroportin, thereby promoting iron retention and sustaining neuroinflammation (117). This “high uptake–low efflux” iron metabolic configuration may transform microglia into pathological iron reservoirs within the white matter, providing a sustained catalytic substrate for lipid peroxidation.

Within this framework, autophagy has been proposed as an upstream regulatory system that may link iron metabolic dysregulation to ferroptosis (118). A growing body of evidence suggests that ferroptosis may be mechanistically conceptualized as a form of autophagy-dependent cell death (56), the execution of which is contingent upon the selective autophagic degradation of key intracellular substrates (119–121). Among the relevant mechanisms, ferritinophagy mediated by nuclear receptor coactivator 4 (NCOA4) occupies a central role. NCOA4 selectively recognizes ferritin and directs its lysosomal degradation, thereby liberating substantial quantities of free iron that directly fuel lipid peroxidation cascades (122). This pathway represents an important molecular mechanism through which iron metabolic dysregulation may promote ferroptosis. In a cerebral ischemia-reperfusion injury model, for instance, upregulation of NCOA4 in conjunction with downregulation of FTH1 has been shown to promote free iron accumulation, oxidative stress, and ferroptosis-associated injury; conversely, silencing of NCOA4 significantly attenuates neuroinflammation (123). In parallel, lipophagy contributes to susceptibility to ferroptosis by degrading lipid droplets and releasing free fatty acids, thereby supplying substrates for lipid peroxidation. Chronophagy, by contrast, has been proposed to amplify oxidative stress at the transcriptional level by modulating the nuclear receptor subfamily 1 group D member 1 (NR1D1)–HO-1 axis, which in turn promotes iron overload.

Within the white matter microenvironment, microglial homeostasis requires efficient autophagy–lysosomal function, particularly during prolonged exposure to myelin-derived lipid debris. When autophagic function is impaired, accumulation of lipid peroxidation substrates and increased oxidative stress may interact with iron retention, thereby reducing antioxidant capacity and increasing susceptibility to ferroptosis. Notably, under specific conditions, activation of the autophagy–lysosomal pathway has been shown to enhance microglial lipid metabolism and attenuate WMI, suggesting that autophagy exerts a context-dependent, dual regulatory role in determining microglial fate.

Collectively, current evidence suggests that autophagy may play a role in regulating microglial iron homeostasis, and a potential pathway involving autophagy-dependent iron retention and lipid peroxidation has been implicated in ferroptosis. Whether this axis drives microglial ferroptosis in WMI and whether it represents a viable therapeutic target warrant further investigation.

### 4.2 Lipid peroxidation amplifiers: the cooperative network of ACSL4, NOX, and LOX

Lipid peroxidation represents a central event in ferroptosis (124), driven by ROS and reactive nitrogen species (RNS), which initiate chain oxidation reactions of PUFAs and ultimately generate phospholipid hydroperoxides (PL-OOH) (125). In microglia, ACSL4 (37), NADPH oxidase 2 (NOX2) (126), and arachidonate 15-lipoxygenase (15-LOX) (127) form a coordinated regulatory network that may collectively function as a “lipid peroxidation amplifier” (128).

ACSL4 is a key positive regulator of ferroptosis execution (129), distinguished by its high substrate specificity for PUFAs (130–132). ACSL4 catalyzes the conversion of PUFAs to polyunsaturated fatty acyl-CoA (PUFA-CoA), which is subsequently esterified by lysophosphatidylcholine acyltransferase 3 (LPCAT3) and incorporated into membrane phospholipids to form highly oxidation-prone polyunsaturated fatty acid-containing phospholipids (PUFA-PL) (133, 134), thereby generating the principal substrate pool for lipid peroxidation (135, 136). In microglia, knockdown of ACSL4 has been shown to reduce susceptibility to ferroptosis (37) and attenuate pro-inflammatory mediator production, collectively contributing to alleviation of ischemic brain injury (137).

NOX2 represents a principal source of ROS in microglia under pathological conditions (138). Upon inflammatory stimulation by triggers such as Aβ or damage-associated molecular patterns (DAMPs), NOX2 acts in concert with the membrane-translocated cytosolic subunits p47^phox and p67^phox to sustain the continuous generation of highly cytotoxic species (139), including superoxide anion (O_2_•^−^), hydrogen peroxide (H_2_O_2_), •OH, and RNS (140). These oxidants collectively provide the redox driving force underlying the Fenton reaction and lipid peroxidation (141). Furthermore, NOX2 participates in a self-reinforcing amplification loop through the activation of Toll-like receptor 4 (TLR4) and complement receptors (142), thereby compounding the risk of WMI and susceptibility to ferroptosis. Consistent with this mechanistic model, NOX2 knockout mice have been shown to significantly reduce Fe^2+^-induced ROS production and associated neurotoxicity in microglia (143).

As an iron-dependent dioxygenase, 15-LOX directly catalyzes the oxidation of PUFA-PLs into lethal lipid peroxidation products—15-hydroperoxyeicosatetraenoic acid-phosphatidylethanolamine (15-HpETE-PE) — thereby functioning as a critical mechanistic link between iron overload and membrane lipid damage (144, 145). Its enzymatic activity is subject to negative regulation by nitric oxide (NO) derived from inducible nitric oxide synthase (iNOS) (146). In oxygen-glucose deprivation and spinal cord injury models, iNOS expression levels have been shown to directly govern microglial susceptibility to ferroptosis (147, 148). Accordingly, targeted inhibition of 15-LOX-1 has been demonstrated to reduce PL-OOH accumulation, thereby conferring protection against ferroptosis-mediated brain injury (146, 149).

Taken together, ACSL4, NOX2, and 15-LOX may act as key upstream components of a “lipid peroxidation amplifier” that increases microglial susceptibility to ferroptosis (150). Their coordinated actions may promote lipid peroxidation beyond endogenous antioxidant capacity, thereby increasing susceptibility to ferroptotic injury.

### 4.3 Multilayered defense system: the system Xc^−^–GSH–GPX4 axis and its backup mechanisms

The cystine/glutamate antiporter System Xc^−^–glutathione (GSH)–glutathione peroxidase 4 (GPX4) axis constitutes a primary defense system against ferroptosis in microglia (151, 152). System Xc^−^, a heterodimeric transporter composed of solute carrier family 7 member 11 (SLC7A11) and solute carrier family 3 member 2 (SLC3A2), sustains GSH biosynthesis through the uptake of extracellular cystine (153). GPX4 uses GSH as a co-substrate to reduce toxic lipid hydroperoxides (L-OOH) to their non-toxic alcohol counterparts (L-OH), thereby limiting lipid peroxidation chain reactions (154); GPX4 further limits the oxidation of PUFAs by suppressing LOX activity (155). This central defense axis is subject to precise transcriptional and epigenetic regulation. Enhancer of zeste homolog 2 (EZH2) represses SLC7A11 expression via trimethylation of histone H3 at lysine 27 (H3K27me3) (156, 157), while LPS suppresses GPX4 transcription through the AlkB homolog 5 (ALKBH5)–protein arginine methyltransferase 2 (PRMT2)–β-catenin signaling axis, thereby simultaneously impairing both arms of the antioxidant system (158). Compounding this vulnerability, microglia exhibit relatively limited basal erythroid 2-related factor 2 (Nrf2) activity that is insufficiently induced under inflammatory conditions (159), resulting in intrinsically limited GSH synthesis and GPX4 expression and a correspondingly low threshold for antioxidant defense (160). Within the white matter microenvironment, this central axis is consequently highly susceptible to decompensation, increasing microglial susceptibility to ferroptosis (18).

In recent years, non-GPX4-dependent defense mechanisms—most notably the ferroptosis suppressor protein 1 (FSP1)–coenzyme Q_10_ (CoQ_10_)–reduced nicotinamide adenine dinucleotide phosphate (NADPH) system and the dihydroorotate dehydrogenase (DHODH)–ubiquinol (CoQH_2_) system — have emerged as important secondary lines of protection against ferroptosis, with potential relevance to white matter preservation. The FSP1–CoQ_10_–NADPH system is localized to the plasma membrane, where FSP1 reduces CoQ_10_ to trap lipid radicals (161), primarily countering oxidative damage at the membrane–lipid interface (162). In contrast, the DHODH–CoQH_2_ system is anchored to the inner mitochondrial membrane, where it acts in concert with mitochondrial GPX4 to maintain local redox homeostasis and prevent uncontrolled mitochondrial lipid peroxidation (163–165). Notably, however, systematic investigations into the direct roles of FSP1 and DHODH in ferroptosis specifically within white matter microglia remain lacking. Future studies should therefore clarify whether and how these secondary defense mechanisms can compensate for impaired GSH-dependent antioxidant capacity during advanced stages of WMI.

The System Xc^−^–GPX4, FSP1–CoQ_10_–NADPH, and DHODH–CoQH_2_ systems act in different cellular compartments and may provide complementary protection against ferroptosis in white matter microglia. Based on this, we propose a “central axis–compartmental compensation–metabolic support” model. When System Xc^−^–GPX4 activity is reduced, microglia may rely more on FSP1- and DHODH-dependent defenses. However, it remains unclear whether these pathways act in sequence or in parallel. In addition, the roles of FSP1 and DHODH in white matter microglia are still poorly understood. Therefore, this model remains hypothetical and requires direct experimental validation (**Figure 3**; **Table 2**). Failure of these protective networks may, in turn, translate ferroptotic susceptibility into impaired microglial support of myelin repair.

**Figure 3:**
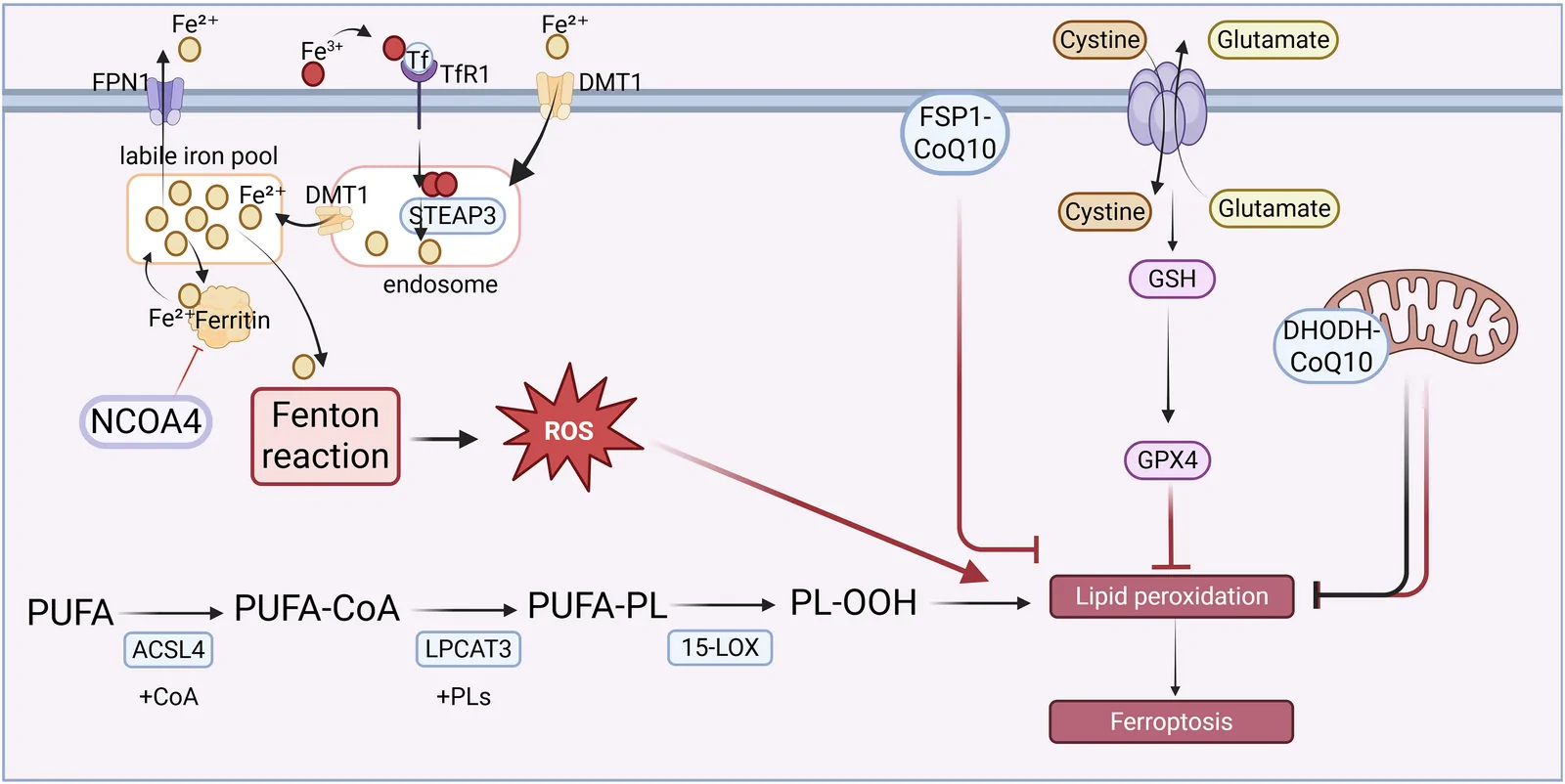
Integrated regulatory networks governing microglial ferroptotic susceptibility. Iron uptake through TfR1-dependent endocytosis, STEAP3-mediated reduction, and DMT1-mediated transport contributes to the expansion of the intracellular labile iron pool, whereas FPN1-mediated iron export limits iron accumulation. NCOA4-mediated ferritinophagy further increases redox-active iron availability by promoting ferritin degradation and iron release. Excess labile Fe^2+^ drives Fenton reaction-mediated ROS generation, thereby promoting lipid peroxidation. In parallel, ACSL4 and LPCAT3 facilitate the formation of oxidation-prone PUFA-PLs, which are further oxidized through 15-LOX-dependent reactions to generate PL-OOH. These pro-oxidant processes are counteracted by the Xc^−^–GSH–GPX4 axis and the complementary FSP1–CoQ_10_ and mitochondrial DHODH–CoQ_10_ (generating CoQH_2_) antioxidant defenses. The balance between iron-dependent oxidative stress, PUFA-PL peroxidation, and antioxidant defenses ultimately determines microglial susceptibility to ferroptosis. 15-LOX, arachidonate 15-lipoxygenase; ACSL4, acyl-CoA synthetase long-chain family member 4; CoQ_10_, coenzyme Q10; CoQH_2_, ubiquinol; DHODH, dihydroorotate dehydrogenase; DMT1, divalent metal transporter 1; Fe^2+^, ferrous iron; FPN1, ferroportin-1; FSP1, ferroptosis suppressor protein 1; GPX4, glutathione peroxidase 4; GSH, glutathione; LPCAT3, lysophosphatidylcholine acyltransferase 3; NCOA4, nuclear receptor coactivator 4; PL-OOH, phospholipid hydroperoxide; PUFA, polyunsaturated fatty acid; PUFA-PL, polyunsaturated fatty acid-containing phospholipid; ROS, reactive oxygen species; STEAP3, six-transmembrane epithelial antigen of prostate 3; system Xc^−^, cystine/glutamate antiporter system; TfR1, transferrin receptor 1. Created with BioRender.com.

**Table 2:** Integrated molecular regulatory networks governing microglial ferroptotic susceptibility in white matter injury.

Pathway	Cell type	Disease/model	Ferroptosis evidence	Intervention/rescue	Functional outcome	Refs.
TfR1–STEAP3–DMT1	Microglia	CNS injury/iron-overload models	Promotes Fe^3+^ uptake and endosomal reduction to Fe^2+^, expanding the labile iron pool and increasing ferroptosis susceptibility	Iron chelation or inhibition of iron uptake	Reduced intracellular iron, oxidative stress, and ferroptosis-associated injury	(81–116)
NCOA4–ferritin (FTH1/FTL)	Microglia	Ischemic/demyelinating CNS injury models	NCOA4-mediated ferritinophagy releases ferritin-bound iron and promotes iron-dependent oxidative stress	Inhibition of ferritinophagy/restoration of ferritin storage	Reduced labile iron and lipid peroxidation; microglia-specific WMI evidence remains limited	(122, 123)
FPN1–hepcidin	Microglia	CNS injury/inflammatory models	Reduced iron export favors intracellular iron retention and increases susceptibility to ferroptosis	Restoration of FPN1-mediated iron efflux/modulation of hepcidin signaling	Reduced iron accumulation and oxidative injury	(100–103)
ACSL4	Microglia	HIV-1 Tat; ischemic brain injury models	Promotes PUFA-PL remodeling and lipid peroxidation; increases ferroptosis susceptibility	ACSL4 knockdown/inhibition	Reduced ferroptosis susceptibility and inflammatory injury	(129–137)
NOX2	Microglia	Fe^2+^/inflammatory stimulation; CNS injury models.	Major ROS source driving oxidative stress and lipid peroxidation	NOX2 knockout/inhibition	Reduced ROS and neurotoxicity; ferroptotic death not independently established	(138–143)
15-LOX	Microglia/related CNS models	OGD, SAH, spinal cord injury	Promotes PUFA-PL oxidation and PL-OOH formation	15-LOX inhibition; iNOS/NO modulation	Reduced lipid peroxidation and ferroptosis-associated injury	(144–149)
System Xc^−^–GSH–GPX4	Microglia	Inflammatory, EAE, and CNS injury models	Loss of GPX4-dependent lipid peroxide detoxification increases ferroptosis susceptibility	Nrf2/SLC7A11/GPX4 restoration	Reduced lipid peroxidation, microglial injury, and inflammation	(151–160)
FSP1–CoQ_10_–NADPH	Mainly non-microglial/general models	General ferroptosis models	GPX4-independent suppression of membrane lipid-radical propagation	FSP1 modulation	Increased ferroptosis resistance; direct white-matter microglial evidence lacking	(161, 162)
DHODH–CoQH_2_	Mainly non-microglial/general models	General/cancer ferroptosis models	Limits mitochondrial lipid peroxidation independently of cytosolic GPX4	DHODH modulation	Increased mitochondrial ferroptosis resistance; direct white-matter microglial evidence lacking	(163–165)

15-LOX, 15-lipoxygenase; ACSL4, acyl-CoA synthetase long-chain family member 4; CNS, central nervous system; CoQ_10_, coenzyme Q_10_; CoQH_2_, ubiquinol; DHODH, dihydroorotate dehydrogenase; DMT1, divalent metal transporter 1; EAE, experimental autoimmune encephalomyelitis; FPN1, ferroportin 1; FSP1, ferroptosis suppressor protein 1; FTH1, ferritin heavy chain 1; FTL, ferritin light chain; GSH, glutathione; GPX4, glutathione peroxidase 4; iNOS, inducible nitric oxide synthase; NADPH, reduced nicotinamide adenine dinucleotide phosphate; NO, nitric oxide; NOX2, NADPH oxidase 2; Nrf2, nuclear factor erythroid 2-related factor 2; OGD, oxygen-glucose deprivation; PL-OOH, phospholipid hydroperoxide; PUFA-PL, polyunsaturated fatty acid-containing phospholipid; ROS, reactive oxygen species; SAH, subarachnoid hemorrhage; SLC7A11, solute carrier family 7 member 11; STEAP3, six-transmembrane epithelial antigen of prostate 3; System Xc^−^, cystine/glutamate antiporter system; TfR1, transferrin receptor 1; WMI, white matter injury.

## 5 Pathological consequences: ferroptosis-associated microglial dysfunction impairs myelin repair

### 5.1 Functional exhaustion: dual loss of microglial phagocytosis and neurotrophic support

Under physiological steady-state conditions, microglia function as important regulators of white matter integrity and plasticity (166). They facilitate the survival, proliferation, and differentiation of OPCs through the efficient clearance of myelin debris and the secretion of critical neurotrophic factors, including insulin-like growth factor-1 (IGF-1) and platelet-derived growth factor (PDGF) (48, 167–169). Data from single-cell lineage tracing and functional knockout models demonstrate that microglial depletion results in the accumulation of myelin debris and impaired OPC recruitment, thereby compromising remyelination efficiency (71).

In WMI, persistent exposure to myelin debris may increase microglial susceptibility to ferroptosis and impair their ability to clear debris (18). Although direct evidence that microglial ferroptosis alone is sufficient to terminate phagocytic activity remains limited, experimental studies have shown that dysfunctional microglial states—characterized by lipid accumulation, altered iron handling, and impaired lysosomal function—exhibit reduced clearance capacity (159). Furthermore, ferroptosis-associated microglial dysfunction may disrupt trophic support and negatively affect OPC survival and maturation capacity (170). Consistent with this, microglial-conditioned medium has been shown to rescue OPCs from death induced by growth factor withdrawal and to promote their differentiation (171). These findings underscore the important contribution of microglial-derived nutritional and metabolic support to effective remyelination.

### 5.2 Secondary injury: direct insult of the toxic microenvironment on the oligodendrocyte lineage

Ferroptosis-associated microglial dysfunction may contribute to a toxic microenvironment through the release of lipid peroxidation products and iron species (172). 4-hydroxynonenal (4-HNE) can induce death of OPCs and mature oligodendrocytes (173) and impair OPC responsiveness to differentiation signals, including platelet-derived growth factor (174). Concurrently, free iron inflicts sustained damage on OPCs and oligodendrocytes via Fenton reactions, constituting a secondary insult. *In vitro* experiments demonstrate that conditioned medium from iron-overloaded microglia induces oligodendrocyte injury, an effect that can be reversed by iron chelators such as deferoxamine (DFO) (175).

### 5.3 Inflammatory amplification: a potential ferroptosis–inflammation feedback loop

Emerging evidence suggests that ferroptosis can modulate immune responses through the release of damage-associated molecular patterns (DAMPs) (176). Current research supports a potential positive feedback model in which DAMP release associated with ferroptosis-associated stress further enhances cellular vulnerability to ferroptosis (177). Ferroptosis-associated cellular stress may promote the release of DAMPs, including high-mobility group box 1 protein (HMGB1), which can activate pattern recognition receptor-mediated inflammatory pathways, particularly the TLR4/receptor for advanced glycation end products (RAGE)–nuclear factor kappa B (NF-κB) axis (178). This activation may engage downstream inflammatory pathways involving NOD-like receptor family pyrin domain-containing 3 (NLRP3)/gasdermin D (GSDMD) and Janus kinase 2 (JAK2)/signal transducer and activator of transcription 3 (STAT3) (179), thereby amplifying neuroinflammatory responses and potentially promoting pyroptotic signaling. In addition, HMGB1 may influence intracellular iron homeostasis through regulation of the hepcidin–ferroportin axis and enhance lipid peroxidation (180), thereby increasing cellular susceptibility to ferroptosis. Collectively, these findings suggest that microglial ferroptosis-associated stress may interact with inflammatory signaling through a potential ferroptosis–inflammation feedback loop, thereby contributing to WMI progression in specific pathological contexts (181, 182) (**Figure 4**). These mechanistic consequences provide the basis for evaluating disease-specific evidence across AD, VaD, and MS.

**Figure 4:**
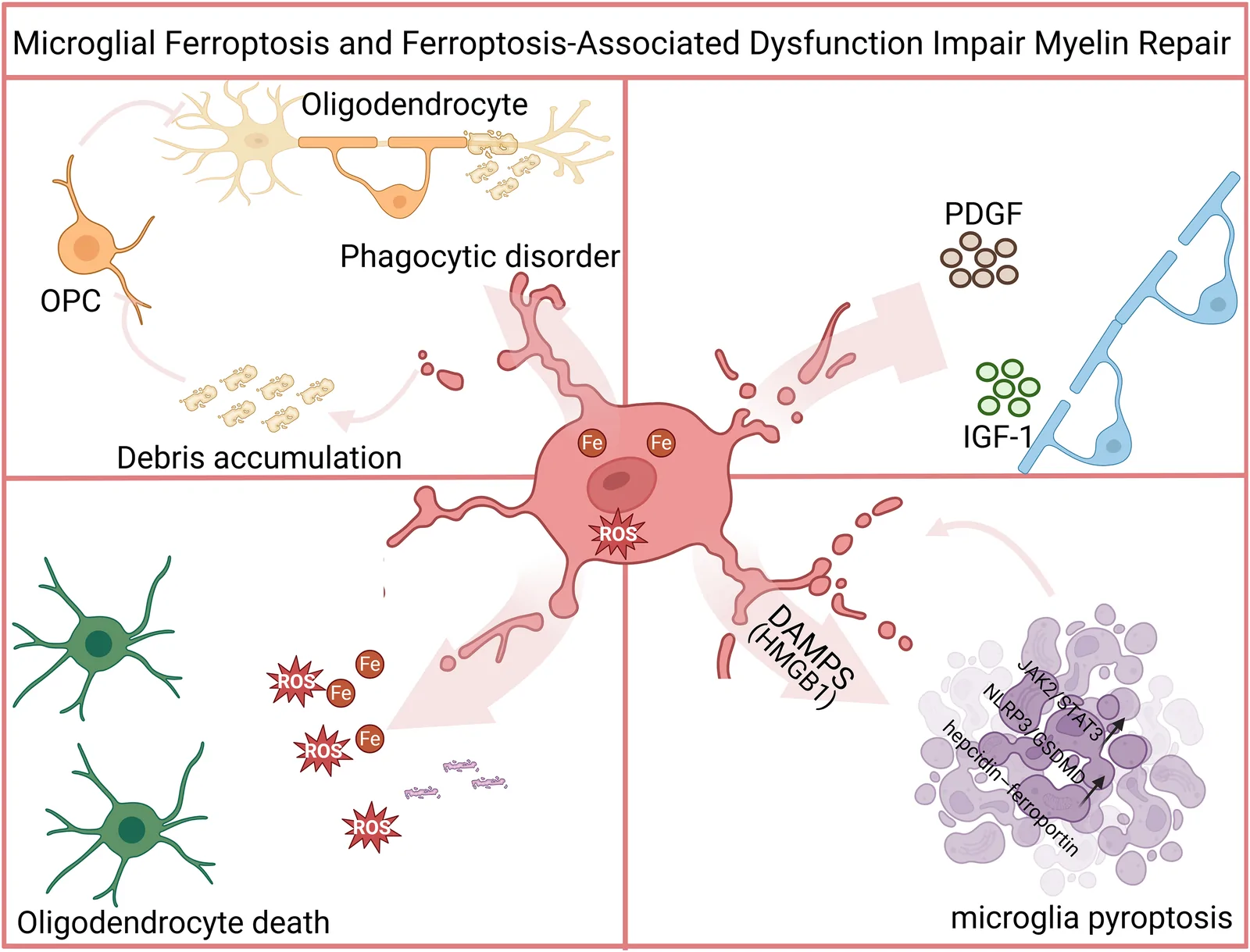
Potential mechanisms by which microglial ferroptosis or ferroptosis-associated dysfunction impairs myelin repair. Microglial ferroptosis or ferroptosis-associated dysfunction may interfere with remyelination through four interconnected mechanisms. First, impaired phagocytic capacity leads to persistent accumulation of myelin debris, thereby limiting oligodendrocyte precursor cell recruitment and differentiation. Second, loss of microglial trophic support reduces the availability of factors such as IGF-1 and PDGF that promote oligodendroglial survival and maturation. Third, redox-active Fe^2+^, ROS, and lipid peroxidation products released from or accumulated around ferroptotic microglia may induce secondary injury to oligodendrocytes and their precursor cells. Fourth, DAMPs, including HMGB1, may amplify neuroinflammation through JAK2/STAT3, NLRP3/GSDMD, and hepcidin–ferroportin signaling, thereby promoting iron retention, inflammatory cell death, and further oxidative injury. Together, these processes may establish a self-amplifying ferroptosis–neuroinflammation loop that contributes to persistent white matter injury and remyelination failure. DAMPs, damage-associated molecular patterns; Fe^2+^, ferrous iron; GSDMD, gasdermin D; HMGB1, high-mobility group box 1; IGF-1, insulin-like growth factor-1; JAK2, Janus kinase 2; NLRP3, NOD-like receptor family pyrin domain-containing 3; OPC, oligodendrocyte precursor cell; PDGF, platelet-derived growth factor; ROS, reactive oxygen species; STAT3, signal transducer and activator of transcription 3. Created with BioRender.com.

## 6 Evidence from disease models

### 6.1 Microglial ferroptosis and ferroptosis-associated dysfunction in Alzheimer’s disease and vascular dementia

AD and VaD represent two predominant forms of dementia, conventionally distinguished by neurodegenerative and vascular pathologies (183), respectively. However, accumulating clinical and pathological evidence demonstrates substantial overlap and interaction between these disorders (184, 185), particularly within white matter pathology. Emerging experimental and neuropathological evidence suggests that microglial ferroptosis may contribute to this shared pathological process and potentially connect white matter injury with cognitive impairment (13).

#### 6.1.1 White matter injury: a cross-pathological marker

White matter hyperintensities (WMHs) constitute a key neuroimaging marker of VaD (186), primarily reflecting white matter lesions, demyelination, and gliosis. The Fazekas score for WMHs demonstrates significant correlation with VaD severity and independently predicts the extent of executive dysfunction and attentional deficits (187). Notably, white matter hyperintensities are also frequently observed in AD patients, suggesting an overlap in the pathological spectrum of these conditions. White matter lesions in AD involve both microvascular pathology and Aβ/tau-mediated degenerative changes, resulting in mixed dementia phenotypes (188). The combined burden of WMHs and Aβ/tau pathology is associated with accelerated cognitive decline (189), indicating that white matter alterations may represent a common pathological convergence point across distinct dementia subtypes (190).

#### 6.1.2 Interaction between vascular and neurodegenerative pathology

The pathological hallmarks of VaD include chronic cerebral hypoperfusion, microvascular lesions, and blood–brain barrier (BBB) dysfunction (191), which collectively restrict oxygen delivery and energy metabolism in white matter regions (192). These perturbations activate local inflammatory cascades and oxidative stress pathways, promoting myelin degradation and white matter structural disintegration. Importantly, BBB disruption occurs early in AD pathogenesis, where it impairs Aβ clearance and facilitates the release of pro-inflammatory mediators, thereby exacerbating white matter damage. Multiple studies have established BBB dysfunction as a critical shared pathological node in both AD and VaD (193). Furthermore, cerebral amyloid angiopathy (CAA)—characterized by Aβ deposition in vessel walls and a pathological hallmark of AD—exhibits striking similarities to the vascular pathology observed in VaD (194).

Beyond its vascular effects, Aβ also engages regulated cell-death pathways that amplify neuronal injury. In entorhinal cortex amyloidopathy, Aβ25–35 induced cognitive deficits alongside ferroptotic and necroptotic alterations; both ferrostatin-1 and necrostatin-1 improved neuropathology and cognition, but only ferrostatin-1 suppressed necroptotic signaling, suggesting ferroptosis may act upstream of necroptosis (195). Consistently, human AD brain shows elevated lipid peroxidation and impaired ferroptosis defenses, while iron chelation with deferoxamine reduces Aβ burden and oxidative lipid damage in ApoEFAD mice (196), pointing to a reciprocal relationship between Aβ pathology and ferroptosis-associated stress—though direct evidence for microglial ferroptosis is still lacking. In summary, Aβ not only promotes the progression of AD by inducing neuroinflammation and disrupting vascular integrity, but may also form a mutually reinforcing pathological loop with ferroptosis-associated oxidative and lipid injury, thereby further exacerbating damage to neural tissue and the disease process (197).

#### 6.1.3 Microglial susceptibility to ferroptosis in white matter injury

WMI represents a common pathological convergence point in both AD and VaD, and microglial ferroptosis has been proposed as a potential cellular mechanism contributing to this convergence. Analysis of post-mortem human brain tissue confirms the presence of abundant degenerative microglia in aged WMI regions of AD and VaD patients. These cells exhibit enrichment of FTL and lipid droplets, along with pronounced lipid peroxidation, mitochondrial stress and DNA damage. They also show elevated expression of ferroptosis-associated gene signatures (13). These features support ferroptosis-associated stress and increased susceptibility to ferroptosis but do not independently establish ferroptotic cell death.

Chronic hypoperfusion, microvascular injury, or Aβ/tau-mediated stress can induce extensive myelin fragmentation (198–201). Concurrently, white matter ischemia and inflammation suppress the Nrf2 pathway in microglia (202, 203), thereby increasing susceptibility to ferroptosis. Conversely, Nrf2 activation can restore iron and lipid metabolic homeostasis (204), inhibit ferroptosis, and promote myelin repair (205).

#### 6.1.4 Reciprocal interactions between white matter injury and cognitive function

As the structural foundation for long-range neural fiber connectivity, WMI contributes to impairments in higher-order cognitive processes, including executive function, information processing speed, and attention (206, 207). Chronic vascular pathology and Aβ/tau-mediated neurodegeneration synergistically compromise white matter integrity in both AD and VaD (193, 208). WMI-associated iron, lipid, and oxidative stress may increase microglial susceptibility to ferroptosis (18). Ferroptosis-associated injury or functional loss of microglia could compromise myelin debris clearance and trophic support and may amplify local inflammation through damage-associated signals, thereby further impairing white matter repair. This concept is supported by emerging evidence from intervention studies. In an Aβ-related AD model, enhanced resistance to ferroptosis in microglia reduced neuroinflammation and improved behavioral outcomes (209), whereas in chronic hypoperfusion-induced WMI, pharmacological or microglia-specific genetic inhibition of ERK5 reduced lipid peroxidation and ferroptosis in microglia, attenuated white matter damage, and improved cognition (80). These findings are consistent with broader evidence linking iron dyshomeostasis, lipid peroxidation, glutathione depletion, and ferroptosis to cognitive dysfunction (195). Thus, microglial ferroptosis and ferroptosis-associated dysfunction may represent a potential convergent mechanism linking AD- and VaD-related white matter pathology to cognitive impairment (13).

#### 6.1.5 The pathological spectrum of mixed dementia

From a pathological perspective, AD and VaD are not entirely discrete entities but may represent overlapping components of a broader pathological spectrum (210). Pure AD or pure VaD are relatively uncommon in clinical practice (211), whereas mixed dementia demonstrates high prevalence in elderly populations (212–214). Mixed dementia reflects the convergence of Aβ/tau pathology and cerebral small vessel disease, with this interaction being particularly evident in white matter abnormalities (213, 215, 216). This pathological interaction may exacerbate WMI while increasing the phagocytic and metabolic burden on microglia, thereby enhancing their susceptibility to ferroptosis.

#### 6.1.6 Summary

Collectively, microglial ferroptosis and ferroptosis-associated dysfunction may represent a context-dependent mechanism linking convergent AD/VaD-related white matter pathology to cognitive dysfunction (**Figure 5**).

**Figure 5:**
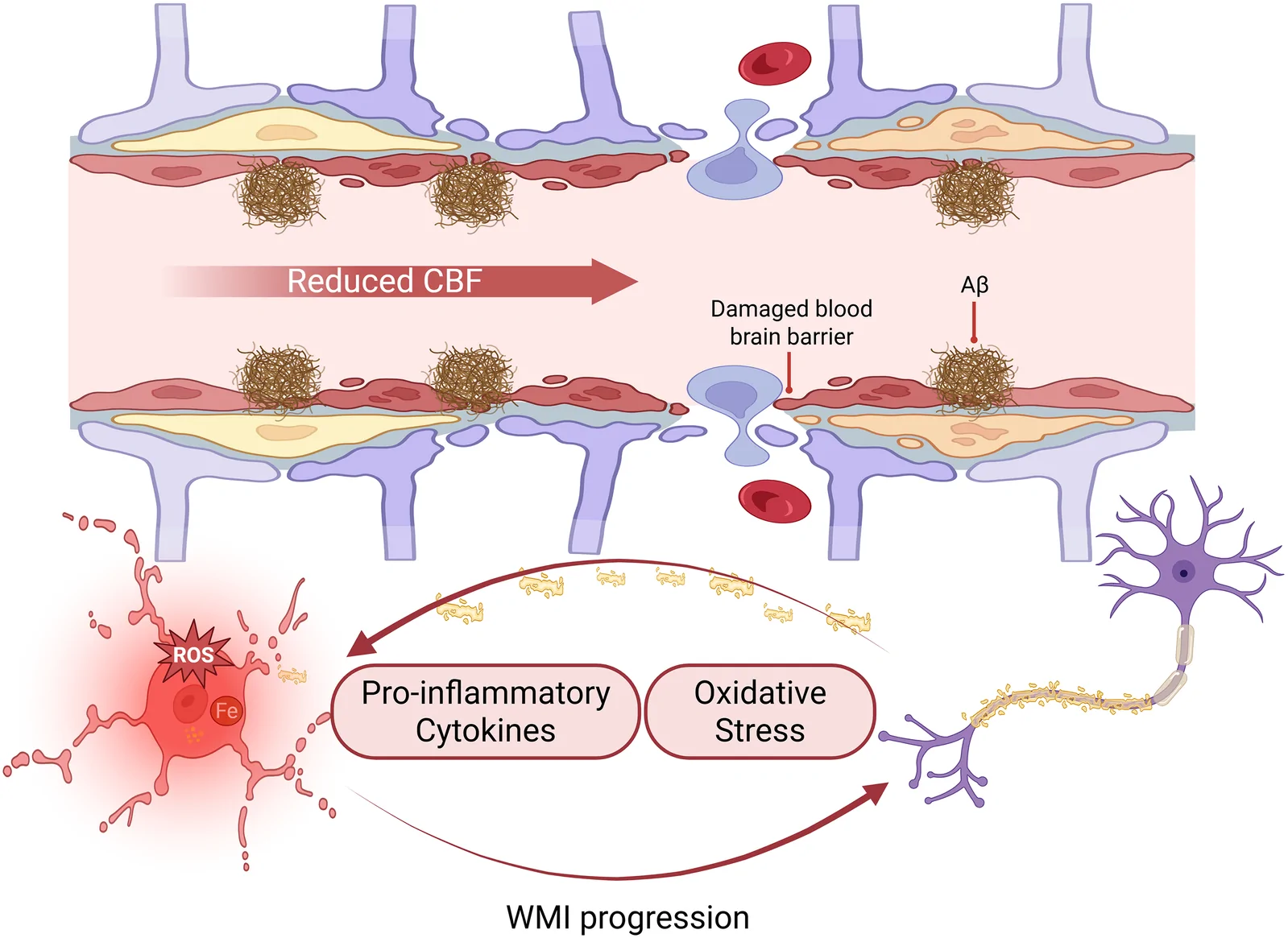
Convergent mechanisms linking microglial ferroptosis to white matter injury in Alzheimer’s disease and vascular dementia. Chronic cerebral hypoperfusion and blood–brain barrier disruption represent major, partially overlapping upstream insults in vascular dementia and Alzheimer’s disease. Blood–brain barrier dysfunction may facilitate the accumulation of circulating and brain-derived neurotoxic mediators and impair amyloid-β clearance, whereas amyloid-β deposition can further compromise vascular and barrier integrity. These interacting processes promote neuroinflammation, oxidative stress, oligodendroglial injury, and myelin degeneration. Persistent uptake of lipid- and iron-rich myelin and cellular debris may then impose phagocytic and metabolic stress on microglia, increasing their susceptibility to ferroptosis. Ferroptotic microglia or microglia with ferroptosis-associated dysfunction may release inflammatory mediators and oxidized lipid products, impair debris clearance, and aggravate secondary injury to oligodendrocytes and myelinated axons. Together, these processes may establish a self-amplifying cycle in which white matter injury increases microglial susceptibility to ferroptosis, while microglial dysfunction further intensifies neuroinflammation, oxidative damage, and remyelination failure, thereby contributing to cognitive decline. Aβ, amyloid-β; BBB, blood–brain barrier; CBF, cerebral blood flow. Created with BioRender.com.

### 6.2 Ferroptosis-associated microglial dysfunction in the pathogenesis of multiple sclerosis

MS is a chronic inflammatory demyelinating disease of the CNS, characterized by focal white matter lesions, progressive neurodegeneration, and cognitive decline. Aberrant microglial activation is a defining pathological feature of MS and a critical driver of neuroinflammatory initiation and perpetuation (217). Emerging evidence indicates that microglia function not only as active “drivers” of inflammation but also as “victims” of the pathological milieu (218). Exposed to iron dysregulation and oxidative stress, microglia may transition from a homeostatic surveillance state toward pro-inflammatory and metabolically stressed phenotypes (219), thereby increasing their susceptibility to ferroptosis. Ferroptosis-associated microglial dysfunction has been proposed as a potential mechanism contributing to MS progression (220).

#### 6.2.1 Iron overload and phagocytic burden: from protective clearance to toxic accumulation

In the pathological environment of MS, substantial quantities of iron released during myelin breakdown are sequestered by microglia, resulting in marked iron accumulation at the margins of lesions and within deep gray matter structures (221). Concurrently, lipid peroxides and oxidatively modified myelin accumulating within MS lesions significantly enhance microglial phagocytic activity (222). Excessive phagocytosis of oxidized myelin has been shown to produce abnormally elevated phagocytic efficiency, increased phagosomal volume, and prolonged phagosomal retention, collectively impairing phagosome maturation and degradation (223). This phagocytic burden not only increases metabolic demands on microglia but also triggers iron metabolism dysregulation, oxidative stress, and autophagy disruption. These alterations may reduce antioxidant capacity and disturb iron handling, thereby creating a cellular environment that increases susceptibility to ferroptosis.

#### 6.2.2 Molecular mechanisms and spatial distribution of ferroptosis-associated microglial dysfunction

As iron accumulation and oxidative stress intensify, microglia may undergo phenotypic and metabolic changes associated with increased susceptibility to ferroptosis. Clinical studies have shown that iron-laden microglia preferentially accumulate at the borders of chronic active white matter demyelinating lesions, where they exhibit pro-inflammatory features (224). These activated cells release substantial quantities of pro-inflammatory cytokines, chemokines, and ROS, establishing a neurotoxic microenvironment that drives axonal injury and lesion expansion (225, 226). Bioinformatic analyses have identified MS-associated ferroptosis-associated genes, including heme oxygenase 1 (HMOX1), LPCAT3, and ribosomal protein L8 (RPL8), whose functions are highly enriched in immune-related and microglial pathways (227–229). Importantly, integrative analysis combining single-cell RNA sequencing, spatial transcriptomics, and spatial proteomics have revealed significant spatial co-localization of microglial activation markers and ferroptosis-associated signatures within MS lesions (230). These findings suggest that ferroptosis-associated microglial dysfunction may represent a relevant pathological process contributing to MS progression through neuroimmune dysregulation and neurodegeneration (220).

#### 6.2.3 Pathological outcome: a central link in the onset and progression of secondary progressive multiple sclerosis

Ferroptosis-associated microglial dysfunction has been implicated in MS progression, particularly during the transition toward secondary progressive MS (SPMS) (219, 224). Microglia experiencing ferroptosis-associated stress or dysfunction may exhibit impaired myelin-debris clearance, while the associated accumulation of iron species and lipid peroxidation products may amplify oxidative stress and inflammatory responses within the surrounding tissue (223). This iron- and lipid-associated feedforward process may contribute to progressive microglial dysfunction and neurodegenerative changes observed during the SPMS stage.

Collectively, these findings suggest that targeting ferroptosis-associated microglial dysfunction and restoring adaptive microglial functions may represent potential therapeutic approaches to limit chronic lesion progression and neurodegeneration in MS (**Table 3**).

**Table 3:** Disease-specific evidence for microglial ferroptosis-associated dysfunction across *in vitro*, animal, and human studies.

Disease context	Evidence level/model	Predominant white matter stressors	Microglial evidence	Potential pathological consequence	Strength/limitation of evidence	Refs.
Alzheimer’s disease	*In vitro*	Inflammation, iron overload, oxidative stress	LPS + FAC induced ROS, iron accumulation, GSH depletion, and reduced Nrf2/GPX4/FTH1 signaling. KMAI@NPs reversed these changes, and the effects were weakened by Nrf2 inhibition.	Reduced ferroptosis-associated stress and preservation of microglial function.	Direct cell-based mechanistic evidence for Nrf2/GPX4-mediated regulation of ferroptosis susceptibility; confirmed ferroptotic cell death was not fully established.	(202)
	Animal	Aβ pathology, neuroinflammation, oxidative stress, iron dyshomeostasis	KMAI@NPs enhanced Nrf2/GPX4/FTH1 signaling, reduced iron accumulation, MDA, and microglial activation, and improved antioxidant capacity.	Reduced Aβ-related injury and neuroinflammation, with improved cognition.	*In vivo* functional evidence linking microglial ferroptosis-associated pathway modulation to improved AD outcomes; microglia-specific ferroptotic death was not directly confirmed.	(202)
	Human	Aβ/tau pathology, cerebral amyloid angiopathy, vascular dysfunction, myelin degeneration, and APOE-associated lipid dysregulation.	Lipid-laden plaque-associated microglia, APOE4-associated lipid-metabolic states, and degenerative microglia with FTL accumulation, lipid droplets, mitochondrial abnormalities, DNA damage, and ferroptosis-associated signatures in white matter lesions.	Impaired debris clearance, persistent lipid storage, inflammatory amplification, and reduced support for remyelination.	Predominantly associative; these features support ferroptosis-associated stress or susceptibility but do not independently confirm ferroptotic cell death.	(13, 64, 67)
Vascular dementia	*In vitro*	Sustained myelin-debris exposure with iron- and lipid-rich substrates.	Myelin-loaded primary microglia show lipid and Fe^2+^ accumulation, mitochondrial oxidative stress, membrane lipid peroxidation, and cytotoxicity preferentially rescued by ferrostatin-1 and deferoxamine; ERK5–NFATc4–CLPTM1L signaling promotes this phenotype.	Loss of metabolic resilience and impaired microglial viability/function.	Direct cellular evidence supporting microglial ferroptosis under myelin-overload conditions, including pharmacological rescue.	(80)
	Animal	Chronic cerebral hypoperfusion, ischemic white matter stress, oxidative injury, and impaired iron–lipid homeostasis.	Pharmacological or microglia-specific genetic inhibition of ERK5 reduces microglial lipid peroxidation and ferroptosis *in vivo*.	Attenuated white matter injury and improved cognitive function.	Provides relatively strong cell-specific causal evidence in experimental WMI, but direct validation across diverse VaD models remains limited.	(80)
	Human	White matter hyperintensities, vascular dysfunction, chronic oxidative stress, myelin loss, and iron–lipid dysregulation.	Degenerative microglia in human AD/VaD white matter lesions exhibit FTL accumulation, PLIN2-positive lipid droplets, mitochondrial abnormalities, DNA damage, and ferroptosis-associated features.	Phagocytic-metabolic decompensation and impaired white matter repair.	Human evidence is mainly pathological and associative; microglia-specific ferroptotic death cannot be inferred from these features alone.	(13, 64)
Multiple sclerosis	*In vitro*	Oxidized myelin, lipid peroxides, and sustained phagocytic burden.	Cell-based models of myelin–microglia interaction show excessive uptake of oxidized myelin, increased phagosomal burden, and impaired phagosome maturation/degradation.	Reduced debris-processing capacity with iron–lipid and oxidative stress.	Supports a mechanistic link between phagocytic overload and ferroptosis-associated vulnerability, but does not alone demonstrate ferroptotic cell death.	(223)
	Animal	Experimental demyelination/EAE, oxidative stress, impaired GPX4 defense, and inflammatory lipid injury.	EAE and demyelination models show reduced GPX4-related defense and experimental modulation of microglial ferroptosis; inhibition of ferroptosis-related pathways can attenuate demyelination and inflammatory injury.	Reduced emyelination/neuroinflammation and improved repair in selected models.	Provides experimental support for microglial ferroptosis in selected animal models, although mechanisms and disease stages are context dependent.	(12, 60, 157)
	Human	Chronic active lesion rims, recurrent demyelination, iron dysregulation, oxidized myelin, and sustained neuroinflammation.	Iron-laden microglia accumulate at chronic active lesion borders; spatial and single-cell studies show co-localization of microglial activation with ferroptosis-associated molecular signatures.	Inflammatory and oxidative amplification, impaired myelin-debris processing, and possible contribution to progressive lesion evolution.	Spatial, transcriptomic, and pathological associations are not equivalent to direct demonstration of microglial ferroptotic death or rescue.	(225, 226, 230)

Aβ, amyloid-β; AD, Alzheimer’s disease; EAE, experimental autoimmune encephalomyelitis; FTL, ferritin light chain; GPX4, glutathione peroxidase 4; MS, multiple sclerosis; PLIN2, perilipin 2; ROS, reactive oxygen species; VaD, vascular dementia; WMI, white matter injury.

## 7 Intervention strategies

Current therapeutic evidence varies across disease models. Some treatments have been tested directly in WMI or demyelination models. Others are supported mainly by studies of related neurological diseases, such as stroke, traumatic brain injury, Parkinson’s disease, and spinal cord injury. These studies provide useful mechanistic or delivery evidence, but their effects on white matter microglia and WMI still require direct validation.

### 7.1 Iron chelating agents

Given that iron dysregulation represents a key component of microglial susceptibility to ferroptosis, iron chelating agents represent a rational therapeutic approach for targeting iron-related pathological processes. The classical iron chelators—deferoxamine (DFO) (231), deferiprone (DFP) (232), and deferasirox (DFX) (233)—were originally developed for the management of systemic iron overload conditions, including β-thalassemia (234), sickle cell disease (235), hereditary hemochromatosis (236), and aplastic anemia (237), and have been increasingly investigated for the prevention and treatment of neurodegenerative diseases over the past two decades (207–209).

Among these agents, DFO is one of the most extensively studied iron chelators (238), with over 30 years of clinical use (239). Experimental studies suggest that DFO can reduce iron accumulation in microglial lysosomal compartments and improve iron homeostasis (175), potentially supporting microglial phagocytic and degradative functions during myelin remodeling (240). Furthermore, DFO has been reported to attenuate microglial senescence-related changes and ferroptosis-associated alterations through modulation of 27-hydroxycholesterol pathways and to reduce age-associated iron accumulation and Aβ metabolic abnormalities in rodent models (241).

### 7.2 Lipid peroxidation inhibitor

Compared with iron chelating agents, direct interruption of the lipid peroxidation chain reaction represents a more targeted mechanistic intervention. Ferrostatin-1 (242) and Liproxstatin-1 (243), both potent radical-trapping antioxidants, intercalate into the phospholipid bilayer to terminate the propagation of lipid peroxyl radicals (244). Myelin debris-induced microglial dysfunction and cell death have been associated with ferroptotic mechanisms, and Ferrostatin-1 has been shown to attenuate these effects and improve ischemic WMI in experimental models (80).

### 7.3 Enhancement of the system Xc^−^–GSH–GPX4 defense axis

Restoration of endogenous antioxidant defenses represents an important strategy for limiting ferroptosis-associated stress, with particular emphasis on modulation of the System Xc^−^/GSH/GPX4 axis (116, 245).

SLC7A11, the subunit of System Xc^−^, is essential for maintaining cellular redox homeostasis and regulating susceptibility to ferroptosis (153). Its transcription is directly regulated by the Kelch-like ECH-associated protein 1 (KEAP1)/Nrf2 pathway (18, 246). For instance, the canonical Nrf2 activator sulforaphane (247) and the novel compound RTA-408 (248) have each been shown to induce SLC7A11 transcription (203), enhance cystine uptake, and consequently augment intracellular GSH biosynthesis (249). In a mouse model of chronic cerebral hypoperfusion with Nrf2 deficiency, aberrant microglial activation was observed together with WMI and accompanied by reductions in antioxidant capacity, including superoxide dismutase (SOD), catalase, and GSH levels, highlighting the importance of this pathway in maintaining redox homeostasis (250).

GPX4 is a central negative regulator of ferroptosis (251), and its enzymatic activity is critically dependent on selenium (252). Selenium enhances GPX4 expression through transcription factor AP-2 gamma (AP-2γ)- and specificity protein 1 (SP1)-dependent transcriptional regulation, thereby increasing cellular resistance to ferroptosis (253). In a murine model of traumatic brain injury, selenium-loaded neural stem cell-derived exosomes (SeNExo) concurrently downregulated pro-inflammatory genes in microglia (including C1qa and Trem2) and ROS levels, while upregulating the remyelination-associated gene Nrg1 in oligodendrocytes, resulting in significant improvement in cognitive outcomes (254) (**Table 4**).

**Table 4:** Therapeutic and translational strategies targeting microglial ferroptosis-associated pathways in white matter injury.

Therapeutic strategy	Representative intervention	Primary mechanism	Reported experimental effect	Major translational limitations	Development stage/refs.
Iron chelation	Deferoxamine; deferiprone; deferasirox	Reduces labile and lysosomal iron, limits Fenton chemistry, and may improve iron handling.	Reduced microglial iron accumulation and ferroptosis-associated changes; support for debris-processing and white matter repair in experimental models.	Non-specific systemic iron depletion, limited target-cell selectivity, BBB delivery, dosing, and long-term safety.	Clinical use for systemic iron overload; neurological application mainly preclinical/exploratory (231–241, 255–257)
Radical-trapping antioxidants	Ferrostatin-1; liproxstatin-1	Interrupt lipid radical propagation within phospholipid membranes.	Attenuation of ferroptosis-associated microglial injury and improvement of experimental ischemic or traumatic WMI.	Poor aqueous solubility/BBB permeability, pharmacokinetic instability, and lack of microglial specificity.	Preclinical (65, 242–244)
System Xc^−^/Nrf2/GSH restoration	Sulforaphane; RTA-408; SLC7A11 induction	Enhances cystine uptake, GSH synthesis, and GPX4-dependent detoxification of lipid hydroperoxides.	Improved antioxidant capacity and reduced microglial activation or ferroptosis-associated injury in experimental models.	Pleiotropic Nrf2 effects, variable cell specificity, and uncertain optimal therapeutic window.	Preclinical (18, 116, 153, 203, 245–250)
GPX4/selenium support	Selenium; selenium-loaded neural stem cell-derived exosomes	Promotes GPX4 expression/activity and strengthens phospholipid hydroperoxide detoxification.	Reduced ROS and pro-inflammatory microglial gene expression, with improved remyelination-associated responses and cognition in animal models.	Dose-dependent selenium toxicity, heterogeneous cargo, manufacturing standardization, and limited disease-specific validation.	Preclinical (251–254)
Targeted nanodelivery	Lipid nanoparticles; PLGA systems; GM@ACSL4-siRNA/NPs	Improves BBB transport and enables delivery of small molecules or gene-silencing cargo to glial targets.	Reduced ferroptosis-associated glial injury and improved neurological outcomes in experimental disease models.	Off-target uptake, scalable manufacturing, biodistribution, immunogenicity, and long-term safety.	Preclinical (258–261)
Engineered exosomes	M2pep-modified adipose-derived stem cell exosomes; SeNExo	Uses endogenous vesicle trafficking and surface targeting to deliver antioxidant or regulatory cargo across the BBB.	Preferential microglial targeting, reduced ferroptosis-associated injury, and improved neurological recovery in experimental models.	Cargo heterogeneity, batch variability, dosing, biodistribution, and uncertain selectivity of M2-like targeting.	Preclinical (254, 262, 263)
AAV-based gene modulation	GPX4/SLC7A11 delivery; GLMP or Alox12 modulation	Sustained manipulation of antioxidant, lysosomal, or lipid-oxidation pathways using selected capsids and microglia-associated promoters.	Cell-targeted modulation of iron deposition, ROS, MDA, and ferroptosis-associated changes in animal models.	Promoter/capsid specificity, peripheral myeloid transduction, irreversible expression, immunogenicity, and species-dependent tropism.	Preclinical (264–269)
Multimodal detection and treatment monitoring	TSPO-PET; SLC7A11-targeted PET tracers; QSM; DTI; blood/CSF biomarkers	Integrates microglial activation, iron-related changes, white matter integrity, and ferroptosis-associated biochemical signals.	Potential stratification of ferroptosis-prone lesions and monitoring of treatment response.	No single modality is ferroptosis-specific; spatial resolution, BBB tracer design, biomarker specificity, and clinical validation remain limiting.	Translational/early development (270–282)

AAV, adeno-associated virus; BBB, blood–brain barrier; CSF, cerebrospinal fluid; DTI, diffusion tensor imaging; GSH, glutathione; GPX4, glutathione peroxidase 4; LNP, lipid nanoparticle; MDA, malondialdehyde; PET, positron emission tomography; PLGA, poly(lactic-co-glycolic acid); QSM, quantitative susceptibility mapping; ROS, reactive oxygen species; TSPO, 18-kDa translocator protein; WMI, white matter injury.

Together, these approaches support the therapeutic potential of targeting iron handling, lipid peroxidation, and antioxidant defense. However, their translation will require improved *in vivo* assessment and better-defined therapeutic windows.

## 8 Future directions

### 8.1 *In vivo* evidence: strategies for dynamic assessment of microglial ferroptosis-associated changes

Current investigations into white matter microglial ferroptosis rely predominantly on static post-mortem tissue analyses (13), precluding dynamic, longitudinal assessment in living subjects—a critical barrier to clinical translation. Establishing a multimodal assessment framework integrating neuroimaging and liquid biopsy may provide a potential strategy for monitoring ferroptosis-associated microglial changes and WMI.

At the molecular imaging level, conventional positron emission tomography (PET) targeting the 18-kDa translocator protein (TSPO) (270, 271) permits quantitative assessment of microglial activation status but is constrained by limited molecular specificity (272, 273). The development of tracers targeting key ferroptosis-related pathways in microglia, therefore, offers considerably greater diagnostic resolution. In this regard, the radiotracer [18F]hGTS13, which targets SLC7A11, has recently been developed for *in vivo* visualization of ferroptosis in a glioblastoma multiforme model (274). Thus, developing BBB-penetrant PET tracers targeting ferroptosis-related pathways, including the SLC7A11/GPX4 axis, represents an important future direction for precision assessment of white matter pathology.

Structural neuroimaging techniques provide essential complementary information at the tissue level. Quantitative susceptibility mapping (QSM) enables highly sensitive quantification of iron deposition in white matter (275), while diffusion tensor imaging (DTI) reflects white matter microstructural integrity through parametric metrics including fractional anisotropy (FA) and mean diffusivity (MD) (276, 277). The combined application of QSM and DTI permits simultaneous evaluation of iron overload and myelin structural integrity (278, 279).

Liquid biopsy offers an accessible and minimally invasive window for systemic biomarker assessment. Iron metabolism indices (e.g., Tf, ferritin) (280) and lipid peroxidation products (e.g., MDA, 4-HNE, PL-OOH) (281) in blood or cerebrospinal fluid may serve as peripheral indicators of altered iron–lipid metabolism and ferroptosis-associated stress in the CNS (282, 283).

Ultimately, integrating multimodal data is essential for precision diagnosis and individualized treatment monitoring. Combining spatiotemporal information on ferroptosis-associated activity, iron deposition, structural damage, and biochemical markers may help localize ferroptosis-related demyelinating lesions. This approach may support early disease detection, evaluation of treatment response, and more accurate definition of therapeutic windows.

### 8.2 A functional therapeutic-window framework

In progressive white matter injury, a major translational challenge is to determine when microglial responses transition from adaptive states toward a state characterized by impaired metabolic resilience and increased susceptibility to ferroptosis (18, 46, 53, 61–63, 66). Current evidence does not support a universal sequential progression from WAM to LDAM and subsequently to degenerative microglia (13, 66, 70). Instead, we propose a functional framework that considers microglial status based on several key parameters, including phagocytic competence, iron–lipid handling capacity, antioxidant reserve, and direct evidence of ferroptotic cell death (61–63). These functional states may coexist and dynamically change depending on the pathological context, rather than representing fixed chronological stages of microglial transformation (13, 66, 70).

#### 8.2.1 Phase I: compensatory phagocytic and metabolic adaptation

During limited or early-stage white matter injury, microglia primarily exhibit adaptive responses characterized by enhanced debris uptake, lysosomal processing, lipid recycling, and antioxidant activation (18, 46, 53, 61–63). Depending on the initiating pathological context, DAM-, DMAM-, or WAM-like programs may be induced; however, no single transcriptional state can be considered a definitive marker of this adaptive phase. Myelin-derived lipids and iron-containing material can be efficiently processed through coordinated mechanisms of degradation, storage, recycling, and export when cellular metabolic capacity remains preserved. Lipid droplets may function as temporary metabolic buffers by sequestering excess oxidizable lipids, provided that lipid turnover, mitochondrial homeostasis, and antioxidant quality-control systems remain intact. Therefore, therapeutic strategies during this phase should focus on limiting upstream vascular or myelin injury while preserving microglial phagolysosomal function, cholesterol efflux capacity, iron sequestration mechanisms, and endogenous antioxidant defenses (18, 46, 53).

#### 8.2.2 Phase II: iron- and lipid-handling decompensation

Sustained debris uptake may eventually exceed the ability of microglia to process lipids and regulate iron, leading to progressive intracellular lipid and iron accumulation (61, 62). At an early stage, microglia may store excess fatty acids in lipid droplets to reduce lipid toxicity (63, 67). However, persistent lipid and iron accumulation, together with metabolic dysfunction and oxidative stress, may indicate reduced metabolic capacity and increased susceptibility to ferroptosis (13, 39, 80, 110, 284). This state may therefore be better assessed using a combination of biomarkers related to lipid metabolism, iron homeostasis, and oxidative stress (127, 285).

In addition to MDA and 4-HNE, oxidized phosphatidylethanolamines, phospholipid hydroperoxides, F_2_-isoprostanes, and neuroprostanes can provide information on lipid peroxidation. Myelin-derived lipids, lipid-storage markers, and markers of fatty acid and cholesterol metabolism may further reflect impaired lipid processing (13, 46, 53, 62, 63, 67, 127, 285–288). However, no single biomarker can confirm microglial ferroptosis. These markers should therefore be interpreted together with measures of iron homeostasis, redox balance, and imaging findings of white matter iron deposition and myelin integrity.

Importantly, this metabolically stressed state may represent a critical therapeutic window, as stressed microglia remain viable and potentially reversible before irreversible cell death occurs. Therapeutic strategies during this phase should aim to restore lipid and iron homeostasis, limit the propagation of membrane phospholipid peroxidation, and enhance endogenous antioxidant capacity.

#### 8.2.3 Phase III: confirmed ferroptotic injury

FTL, PLIN2, γH2AX, TOM20 abnormalities, lipid droplets, iron deposits, and mitochondrial stress are indicative of iron–lipid imbalance, oxidative stress, or cellular injury (13). However, these alterations alone are insufficient to establish the occurrence of ferroptosis (13, 39, 80, 110, 127, 284, 285). To define microglial ferroptosis in white matter, several key criteria should be considered: (1) definitive identification of the affected cells as microglia; (2) evidence of cell death or membrane integrity disruption; (3) accumulation of excessive membrane phospholipid peroxidation; (4) demonstration of iron dependency; and (5) prevention or reversal of cell death by validated ferroptosis inhibitors, iron chelators, or genetic restoration of ferroptosis defense pathways. Whenever possible, alternative forms of regulated cell death, including apoptosis, necroptosis, and pyroptosis, should also be excluded (56, 289, 290).

In human post-mortem tissues, microglial populations exhibiting FTL accumulation, PLIN2 expression, γH2AX-associated DNA damage, or mitochondrial abnormalities should therefore be described as ferroptosis-associated or ferroptosis-prone microglia, unless direct evidence of ferroptotic rescue is available (13, 39, 80, 110, 284). Following extensive microglial loss and secondary tissue damage, the probability of complete functional recovery may decline; however, recovery is not necessarily impossible in all contexts (18, 39, 80, 284). At this stage, therapeutic strategies should focus on preserving surviving microglia and oligodendrocytes, limiting the propagation of oxidized lipids and inflammatory responses, and maintaining the residual capacity for remyelination.

### 8.3 Translational challenges: overcoming the blood-brain barrier, targeted delivery, and safety considerations

Despite promising basic research on ferroptosis in white matter microglia, clinical translation faces three main challenges: delivery efficiency, targeting specificity, and long-term safety.

Because direct evidence for microglia-targeted delivery in WMI remains limited, several of the approaches discussed below are supported primarily by proof-of-concept studies in other neurological disease models and should not be interpreted as established therapeutic strategies for WMI.

A primary obstacle is the BBB, which substantially limits the effective delivery of therapeutic agents to white matter lesions. Many hydrophilic ferroptosis inhibitors—such as ferrostatin-1—exhibit poor BBB permeability, necessitating the development of novel carrier technologies (258). Lipid nanoparticles (LNPs) have shown great promise as carriers for hydrophilic drugs (259, 260). Surface functionalization of LNPs with antibodies or ligands that target microglia-specific receptors can boost brain drug accumulation and reduce off-target effects. In a representative murine model of Parkinson’s disease, a glial membrane (GM)-coated poly(lactic-co-glycolic acid) (PLGA) nanoparticle system delivering ACSL4-targeting small interfering RNA (GM@ACSL4-siRNA/NPs) was shown to attenuate ferroptosis-associated glial injury and improve both motor and cognitive function (261). Engineered exosomes represent a complementary approach, leveraging their inherent biocompatibility and intrinsic capacity to traverse the BBB (262). Notably, M2pep-modified adipose-derived stem cell exosomes (M2pep-ADSC-Exo) developed for ischemic stroke have been shown in both *in vitro* and *in vivo* settings to preferentially target M2-like microglia, attenuate ferroptosis-associated injury, and promote neurological recovery (263).

Gene therapy strategies offer an additional avenue for precise mechanistic regulation. Adeno-associated virus (AAV) vectors are particularly well-suited for this purpose owing to their low immunogenicity and neurotropism (264, 265). Thus, they serve as effective vehicles for delivering antioxidant transgenes such as GPX4 and SLC7A11. The combination of specific AAV serotypes (e.g., AAV9, AAV-PHP.eB) with microglia-specific promoters (e.g., CX3CR1 or F4/80) enables long-term and specific expression of exogenous genes in CNS microglia. In a mouse model of spinal cord injury, microglia-targeted knockdown of glycosylated lysosomal membrane protein (GLMP) using AAV9-Cx3cr1-shGlmp-eGFP led to a significant increase in iron deposition and ferroptosis-associated changes, whereas GLMP overexpression attenuated these effects (266). In a mouse model of Parkinson’s disease, microglia-targeted knockdown of Alox12 using AAV-shRNA driven by the CD68 promoter reduced microglial MDA and ROS levels and attenuated ferroptosis-associated injury (267). Future refinements, including optimization of cell-sorting strategies (268), introduction of targeted capsid point mutations (269), or incorporation of specific nanobodies (244), may further restrict off-target transduction. This thereby provides a genetically precise means of modulating ferroptosis-associated pathways in microglia.

These studies demonstrate the technical feasibility of microglia-targeted ferroptosis modulation, but their efficacy and safety require direct validation in WMI-specific models.

Finally, the pleiotropic nature of ferroptosis warrants careful consideration (136). Beyond its pathological role in cell death, ferroptosis serves essential physiological functions, including cellular homeostasis, immunosurveillance against tumors (291), and clearance of pathogens (292). Consequently, sustained and broadly acting inhibition of ferroptosis carries the risk of adverse outcomes, including impaired antitumor surveillance and weakened host defense (293). This inherent “double-edged sword” characteristic underscores the need for targeted, context-dependent therapeutic strategies rather than systemic ferroptosis suppression (294).

## 9 Conclusion

This article provides an integrative framework for understanding the potential contribution of microglial ferroptosis to WMI. The distinctive iron- and lipid-rich microenvironment of white matter may impose substantial phagocytic and metabolic burdens on microglia, thereby compromising their protective scavenging functions and increasing their susceptibility to ferroptosis. Such dysfunction may interfere with myelin debris clearance, trophic support, and remyelination (18). Accordingly, microglial ferroptosis may represent an important context-dependent immunometabolic mechanism linking white matter injury and associated neurovascular dysfunction to impaired myelin repair, rather than a universally established pathogenic checkpoint.

Despite considerable progress, several fundamental questions remain unresolved. First, the spatiotemporal interplay among ferroptosis, apoptosis, and pyroptosis within white matter lesions requires further clarification. Given the marked heterogeneity of microglia, it also remains unclear which microglial populations are most vulnerable to ferroptosis and which molecular or epigenetic programs determine this susceptibility. Second, definitive *in vivo* evidence of microglial ferroptosis is still limited. Ferroptosis-associated features, including iron accumulation, lipid droplets, oxidative stress, and mitochondrial abnormalities, should be distinguished from bona fide ferroptotic cell death through cell-specific identification, membrane phospholipid peroxidation, iron dependence, and rescue by validated ferroptosis-directed interventions. Third, whether microglial ferroptosis actively drives WMI or instead emerges secondary to metabolic dysfunction remains to be established through temporally resolved and cell-specific studies. Finally, therapeutic strategies must determine whether susceptibility to ferroptosis can be selectively targeted without compromising the beneficial functions of microglia in debris clearance, trophic support, and tissue repair. Resolving these issues will be critical for defining therapeutic windows and enabling the rational translation of ferroptosis-targeted interventions in white matter disease.
